# Improving the Quantification of DNA Sequences Using Evolutionary Information Based on Deep Learning

**DOI:** 10.3390/cells8121635

**Published:** 2019-12-14

**Authors:** Hilal Tayara, Kil To Chong

**Affiliations:** 1 Department of Electronics and Information Engineering, Chonbuk National University, Jeonju 54896, Korea; hilaltayara@jbnu.ac.kr; 2 Advanced Electronics and Information Research Center, Chonbuk National University, Jeonju 54896, Korea

**Keywords:** convolution neural network, deep learning, DNA computing, evolutionary information, LSTM, non-coding DNA

## Abstract

It is known that over 98% of the human genome is non-coding, and 93% of disease associated variants are located in these regions. Therefore, understanding the function of these regions is important. However, this task is challenging as most of these regions are not well understood in terms of their functions. In this paper, we introduce a novel computational model based on deep neural networks, called DQDNN, for quantifying the function of non-coding DNA regions. This model combines convolution layers for capturing regularity motifs at multiple scales and recurrent layers for capturing long term dependencies between the captured motifs. In addition, we show that integrating evolutionary information with raw genomic sequences improves the performance of the predictor significantly. The proposed model outperforms the state-of-the-art ones using raw genomics sequences only and also by integrating evolutionary information with raw genomics sequences. More specifically, the proposed model improves 96.9% and 98% of the targets in terms of area under the receiver operating characteristic curve and the precision-recall curve, respectively. In addition, the proposed model improved the prioritization of functional variants of expression quantitative trait loci (eQTLs) compared with the state-of-the-art models.

## 1. Introduction

High throughput sequential data availability has attracted researchers to develop outstanding deep learning algorithms that can efficiently learn from large datasets [1]. Take into consideration that over 98% of the human genome is non-coding regions, and the function of these regions is not very well understood. Thus, having a computational model that can predict the function of the non-coding DNA region from raw genomic data is important. A genome-wide association study revealed over 6500 trait predisposing single nucleotide polymorphisms (SNPs) or diseases where 93% of these diseases or SNPs are located in the non-coding regions [2]. This illustrates the significance of developing predictive computational tools for understanding the functionality of non-coding regions in DNA.

Recent research aimed to predict the function of the genomic sequences directly from the raw genomics data instead of handcrafting the features. In this regard, deep learning algorithms have produced remarkable results as they are able to learn automatically complex patterns from large datasets. Deep learning has been applied successfully to a wide range of problems such as image and sound processing [3,4,5,6], natural language processing [7], machine translation [8], and various computational biology tasks [9,10,11,12,13,14,15].

Recently, the function of the non-coding DNA regions was studied by Zhou and Troyanskaya [16] and Quang and Xie [17]. Zhou and Troyanskaya proposed the DeepSEA model in which they utilized the convolution neural network for capturing the important motifs from the raw DNA sequences. Their proposed model was simple as it contained three consecutive convolution layers followed by fully connected layers for classification. This model was improved by Quang and Xie by proposing the DanQ model. The DanQ model improved DeepSEA by adding a recurrent layer in order to capture the dependencies between the learned motifs of the convolution layers. Therefore, the DanQ model outperformed DeepSEA one.

In this paper, we improve the aforementioned models by presenting a new deep learning design and adding evolutionary information to the raw genomic sequences. The proposed model consists of two identical networks with shared weights for dealing with input sequences for forward and reverse complement DNA strands. In this work, we train the proposed model by using the datasets prepared by DeepSEA and also used by DanQ. It contains 690 transcription factor binding profiles for 160 different transcription factors, 104 histone marker profiles, and 125 DNase I hypersensitive sites’ profiles. This results in having 919 targets for each input sequence. The proposed deep learning model DQDNNwas trained using raw DNA sequences only, and we call it DQDNN-DNA; and by integrating the evolutionary information (conservation scores) with DNA sequences, we call it DQDNN-CONS. The evaluation results show that the proposed model outperforms the current state-of-the-art models by using raw DNA sequences only and also by integrating evolutionary information with raw DNA sequences. Furthermore, the prioritization of functional variants of expression quantitative trait loci (eQTLs) was improved compared with the state-of-the-art models.

## 2. Materials and the Proposed Models

### 2.1. Materials

In this work, we used the dataset prepared by the DeepSEA model and used by the DanQ model [16,17]. This dataset was prepared by dividing the genome into 200 bp subsequences, and for each subsequence, 919 chromatin features were computed as labels. The input sample was prepared by taking 1000 bp centered on the 200 bp subsequences from the GRCh37/hg19 reference genome assembly. The testing dataset was prepared from chromosome 8 and chromosome 9 with 275,000 sequences. The validation dataset was prepared from chromosome 7 with 4000 sequences. The remaining chromosomes were used for the training dataset with 2,200,000 sequences. The areas under the precision-recall and receiver operator curves were used to evaluate the performance of the proposed model, and since the dataset was imbalanced, the area under the precision-recall curve was the most important metric that accurately described the performance of the proposed model.

### 2.2. The Proposed Model

**Figure 1:**
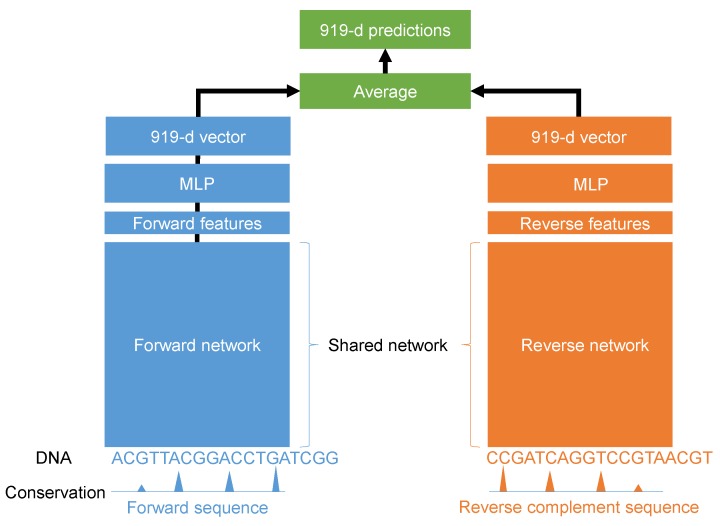
Illustration of the proposed deep learning model DQDNN.

In this paper, we propose a deep learning model for the quantification of non-coding DNA regions. Instead of treating forward and reverse complement sequences independently, we considered using both sequences together as the input. The proposed model is illustrated in Figure 1. It was a Siamese architecture in which the weights of the forward and reverse complement networks were shared. Different architectures were tested using the grid search algorithm. For every input sequence S with L nucleotide, we encoded A, G, C, and T using the one-hot method such as A being represented by [1 0 0 0], G being represented by [0 1 0 0], C being represented by [0 0 1 0], and T being represented by [0 0 0 1]. In this work, L is 1000 nucleotides. In addition, we added the evolution score for every nucleotide in the input sequence. Therefore, the final input would have a shape of 1000×5 such that four channels were for one-hot encoding and the last channel was for the evolution scores.

**Figure 2:**
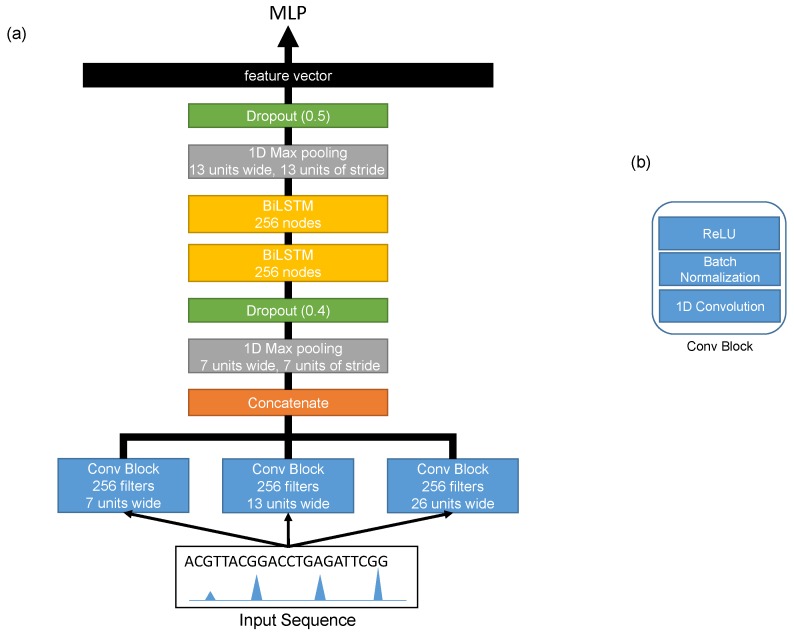
The detailed architecture of the forward/reverse complement network (**a**). The configurations of the Conv block (**b**).

The architecture of the shared network is shown in Figure 2. It consisted of three convolution layers running in parallel in order to extract different features (motifs) at different scales from the input sequences. Each convolution layer was a one-dimensional convolution layer [18] with 256 filters, and the sizes of the filters of these layers were 26, 13, and 7. Each convolution layer was followed by a batch normalization layer [19] and a rectified linear unit (ReLU) [20]. The outputs of these convolution layers were then concatenated and passed threw a max-pooling layer with a window size of 7 and a stride of 7. Then, a dropout layer [21] was added with a probability of 0.4. After that, we added two bidirectional LSTM layers [22] with 256 nodes in order to extract the long term relationships between the extracted features from the first convolution layers. The output of the second bidirectional LSTM layer went through a max-pooling layer with a window size of 13 and a stride of 13 and a dropout layer with a probability of 0.5. The final output was flattened into a feature vector representing the learned features of the input sequence. The detailed configurations are shown in Table 1.

**Figure 3:**
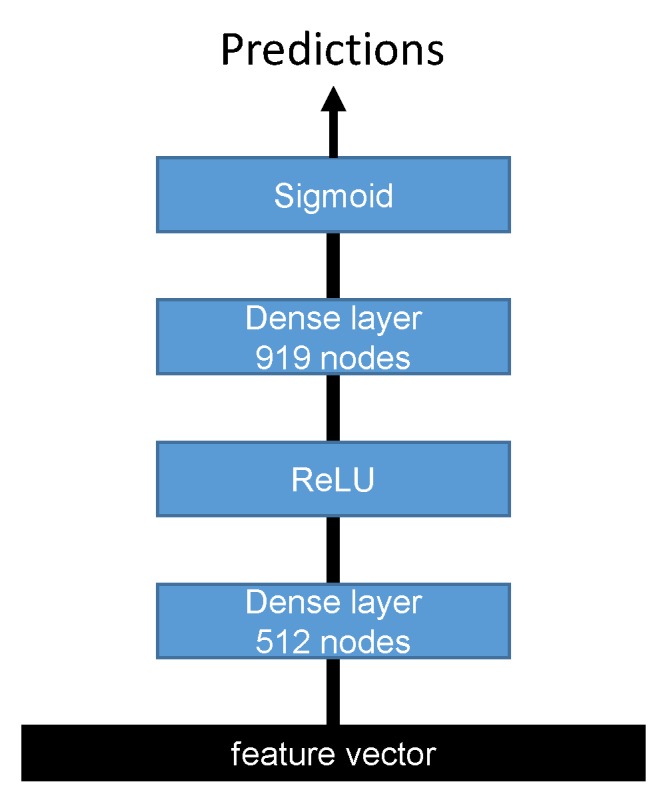
The detailed architecture of the MLP classifier.

Two feature vectors were extracted from forward and reverse complement networks, and each one was passed to a multi-layer perceptron (MLP) network with two dense layers, as shown in Figure 3.

The first dense layer had 512 nodes followed by the ReLU activation function, while the second dense layer had 919 nodes with a sigmoid activation function. Finally, the outputs of the second fully connected layer of the forward and reverse complement inputs were averaged to output the final predictions. The detailed configurations of the MLP classifier are shown in Table 2.

In Table 1, the operation Conv1D(f,s,t) is a one-dimensional convolution layer with *f* filters of size *s* and stride *t*. It can be expressed mathematically by Equation (1) where *X* is the input feature map and *i* and *k* are the indices of the output position and the kernels, respectively. $W^{k}$ is a convolution kernel with an M×N weight matrix of a window size of *M* and a number of input channels of *N*.

(1) $$\mathrm{Conv}{\left(X\right)}_{ik}=\mathrm{ReLU}\left(\sum_{m=0}^{M-1}\sum_{n=0}^{N-1}W_{mn}^{k}I_{i+m,n}\right)$$

The operation “Concatenate” links together all outputs from the three convolution layers. The operation Max_pooling_1D(W,t) is a pooling function that selects the maximum value within a window *W* and stride *t*. It is expressed mathematically in Equation (2), where *X* is the input and *i* and *k* represent the indices for output position and the kernels, respectively.

(2) $$\mathrm{Max}\_\mathrm{pooling}\_1D{\left(X\right)}_{ik}=max\left(\left\{X_{iW,k},X_{(iW+1),k},...,X_{iW+W-1,k}\right\}\right)$$

The Dropout(α) operator drops some nodes with a probability of α at training time in order to avoid over fitting. The operator BiLSTM is a bidirectional long short term memory that helps in capturing the dependencies among the learned motifs of the first layers. Thus, considering an input sequence {**x**}${}_{t=1}^{T}$, the LSTM has cell states {**C**}${}_{t=1}^{T}$ and hidden states {**h**}${}_{t=1}^{T}$ and outputs a sequence {**o**}${}_{t=1}^{T}$. This can be expressed mathematically by Equation (3) where $W_{i}$, $W_{o}$, $W_{f}$, $U_{i}$, $U_{o}$, and $U_{f}$ are the weight matrices and $b_{o}$, $b_{c}$, $b_{i}$, and $b_{f}$ are the biases. Sigmoid and Tanh are the activation functions.

(3) $$\begin{array}{cc} f_{t} & =\sigma (b_{f}+U_{f}h_{t-1}+W_{f}x_{t})\\ i_{t} & =\sigma (b_{i}+U_{i}h_{t-1}+W_{i}x_{t})\\ c_{t} & =i_{t}⊙tanh\left(b_{c}+U_{c}h_{t-1}+W_{c}x_{t}\right)+f_{t}⊙c_{t-1}\\ o_{t} & =\sigma (b_{o}+U_{o}o_{t-1}+W_{o}x_{t})\\ h_{t} & =o_{t}⊙tanh\left(c_{t}\right) \end{array}$$

The Flatten operator converts the learned features from a 2D vector to a 1D vector to be used in the fully connected layers. In Table 2, Dense(*n*) is a fully connected layer with *n* nodes, and the output of each node is described mathematically as:

(4) $$f=w_{d+1}+\sum_{k=1}^{d}w_{k}z_{k}$$

where *z* is the incoming 1D vector, $w_{k}$ is the weight of $z_{k}$’s contribution to the output, and $w_{d+1}$ is the additive bias term. ReLU and Sigmoid are nonlinear activation functions and described in Equations (5) and (6), respectively, where *z* represents the input to these functions.

(5) ReLU(z)=max(0,z)

(6) $$\mathrm{Sigmoid}\left(z\right)=\frac{1}{1+e^{-z}}$$

The proposed model was designed and implemented by the Keras deep learning framework (https://keras.io/). The Adam optimizer [23,24] was used with a learning rate of 0.001 and a batch size of 1000 divided on 4 TitanXP GPUs. The number of training epochs was set to 60. The evolutionary information was obtained from http://hgdownload.cse.ucsc.edu/goldenpath/hg19/phyloP100way/, where we used the conservation scores of multiple alignments of 99 vertebrate genomes to the human genome. These scores were obtained from the Phylogenetic Analysis with Space/Time Models (PHAST) package (http://compgen.bscb.cornell.edu/phast/). For performance evaluation, we followed [16,17] by using the area under the operating receiver curve (ROC-AUC) and the area under the precision-recall curve (PR-AUC). The PR-AUC was more important than ROC-AUC as the dataset we used for evaluation was imbalanced [25].

### 2.3. Functional SNP Prioritization

**Figure 4:**
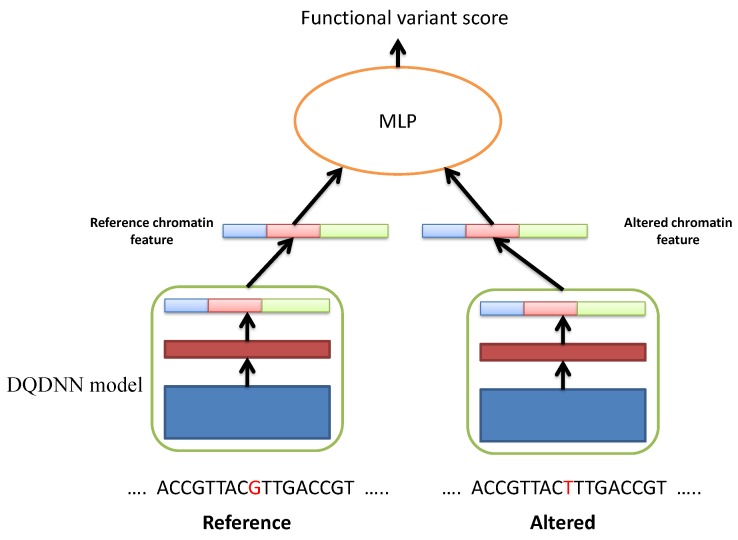
Illustration of the functional SNP prioritization model.

The proposed DQDNN model could be used to study the functional SNP prioritization. Here, we used the positive and negative datasets provided by DeepSEA. The positive dataset was obtained from the genome-wide repository of associations between SNPs and phenotypes (GRASP) database, and it includes the expression quantitative trait loci (eQTLs) [26]. For the negative dataset, we used 1000 Genomes Project SNPs [27]. The negative SNPs dataset was divided into different groups based on their distances to the positive standard SNPs such as 360 bp, 710 bp, 6.3 kbp, and 31 kbp. By following DeepSEA and DanQ, the features for the positive and negative SNP sequences were extracted using the proposed model DQDNN. Then, these features were passed to a multi-layer perceptron (MLP) neural network to learn the functional SNP prioritization as shown in Figure 4.

In more detail, we extracted the chromatin features using DQDNN for the reference sequence, and we call it $f_{ref}$; and the altered sequence we call $f_{alt}$. From these two chromatin features vectors, we calculated 2×919 chromatin effect features that were the concatenation of the absolute differences:

(7) $$|f_{ref}-f_{alt}|$$

and the relative log fold changes of odds:

(8) $$|\log\frac{f_{ref}}{1-f_{ref}}-\log\frac{f_{alt}}{1-f_{alt}}|$$

In our design, we calculated the chromatin effect features from DQDNN-DNA and DQDNN-CONS. Thus, we had 2×2×919=3676 chromatin effect features to be used in the MLP model. The MLP model was a two layer fully connected network, and the detailed configurations of the MLP model are given in Table 3.

## 3. Results and Discussion

### 3.1. The Performance of the DQDNN Model

The proposed deep learning model DQDNN was trained using raw DNA sequences only, and we called it DQDNN-DNA; and by integrating the evolutionary information (conservation scores) with DNA sequences, we called it DQDNN-CONS. The results showed that adding evolutionary information improved both average ROC-AUC and average PR-AUC, as given in Table 4. The results showed that adding conservation scores improved the performance by 1.97% in terms of average PR-AUC.

**Figure 5:**
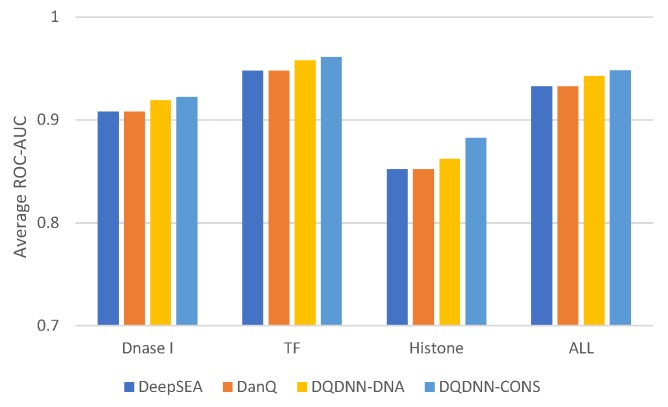
The average ROC-AUC comparison of the proposed model with the state-of-the-art models.

**Figure 6:**
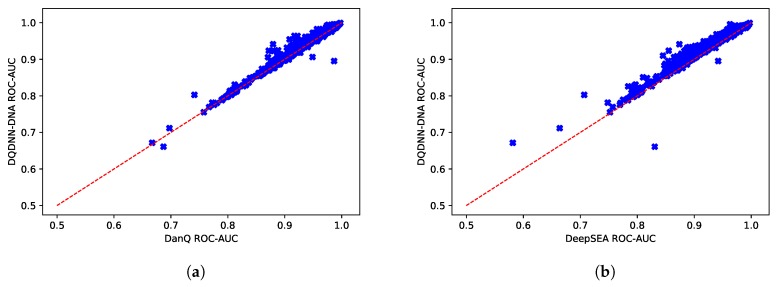
Scatter plot comparing the ROC-AUC scores of the proposed model DQDNN-DNA and (**a**) DanQ and (**b**) DeepSEA models.

**Figure 7:**
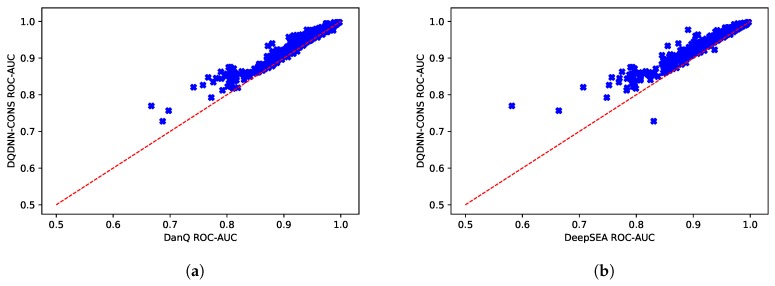
Scatter plot comparing the ROC-AUC scores of the proposed model DQDNN-CONS and (**a**) DanQ and (**b**) DeepSEA models.

Furthermore, we evaluated the performance of the proposed system with the state-of-the-art models, namely DanQ [17] and DeepSEA [16]. Table 5 and Figure 5 show the comparison of the average ROC-AUC of the proposed model DQDNN with the DanQ and DeepSEA models. In more detail, DQDNN-DNA performed better than DanQ in 85.4% of the targets (785 out of 919), as shown in Figure 6a, and performed better than DeepSEA in 98% of the targets (901 out of 919), as shown in Figure 6b. On the other hand, the integration of conservation scores (DQDNN-CONS model) improved the performance in 96.9% of targets (891 out of 919) and 99.3% of targets (913 out of 919) compared to DanQ (Figure 7a) and DeepSEA (Figure 7b), respectively.

**Figure 8:**
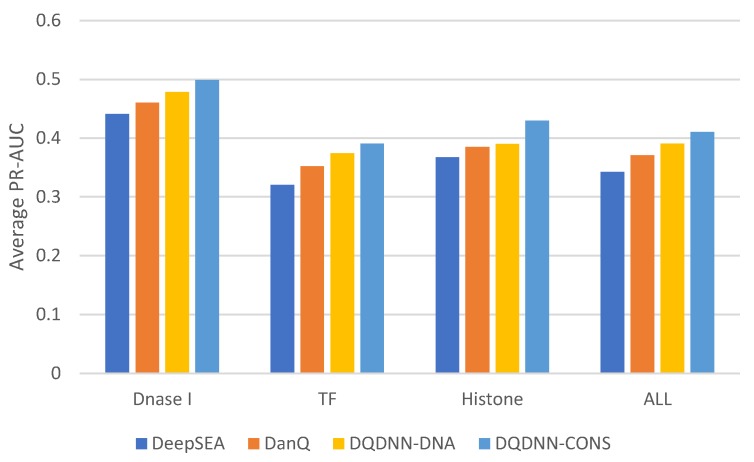
The average PR-AUC comparison of the proposed model with the state-of-the-art models.

**Figure 9:**
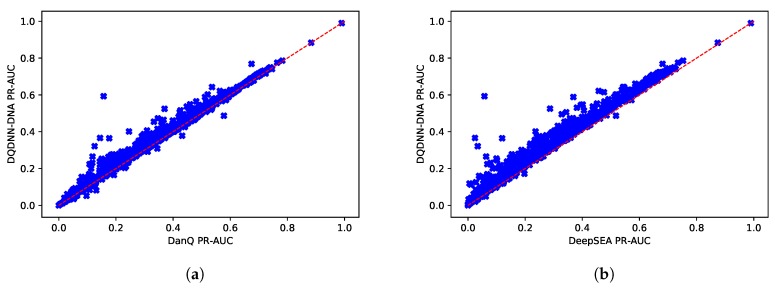
Scatter plot comparing the PR-AUC scores of the proposed model DQDNN-DNA and (**a**) DanQ and (**b**) DeepSEA.

**Figure 10:**
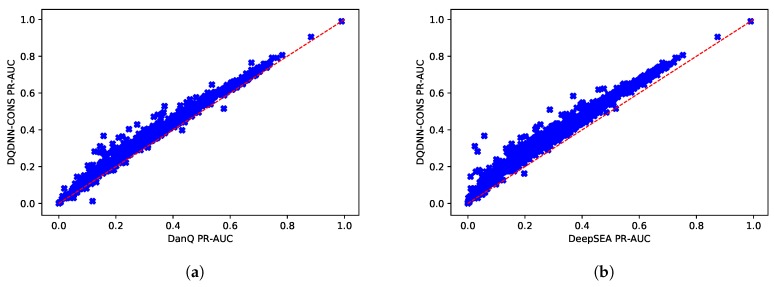
Scatter plot comparing the PR-AUC scores of the proposed model DQDNN-CONS and (**a**) DanQ and (**b**) DeepSEA.

Since the dataset was imbalanced, the PR-AUC was a more expressive metric than ROC-AUC. Table 6 and Figure 8 show the comparison of the average PR-AUC of the proposed model DQDNN with the DanQ and DeepSEA models. In more detail, DQDNN-DNA performed better than DanQ in 88% of the targets (809 out of 919), as shown in Figure 9a, and performed better than DeepSEA in 98% of the targets (903 out of 919), as shown in Figure 9b. On the other hand, the integration of conservation scores (DQDNN-CONS model) also improved the performance in 98% of targets (898 out of 919) and 99.1% of targets (911 out of 919) compared to DanQ and DeepSEA, as shown in Figure 10a and Figure 10b, respectively.

**Figure 11:**
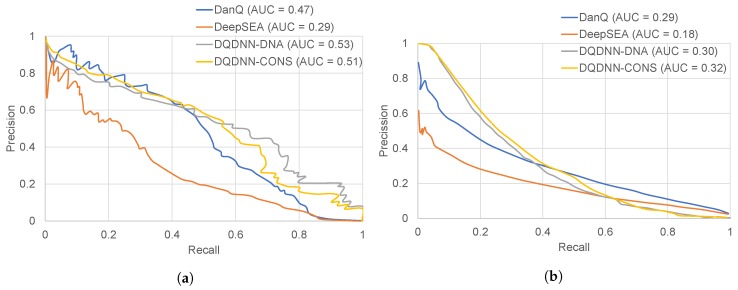
Examples of the PR-AUC comparison of the proposed model DQDNN with DanQ and DeepSEA for (**a**) H1-hESC SIX5 and (**b**) GM12878 EBF1.

The detailed ROC-AUC and PR-AUC for all 919 targets are given in Supplementary File 1. For example, we show in Figure 11 the PR-AUC for the GM12878 EBF1 and H1-hESC SIX5 of the DQDNN, DanQ, and DeepSEA models.

### 3.2. The Performance of the Functional SNP Prioritization Model

**Figure 12:**
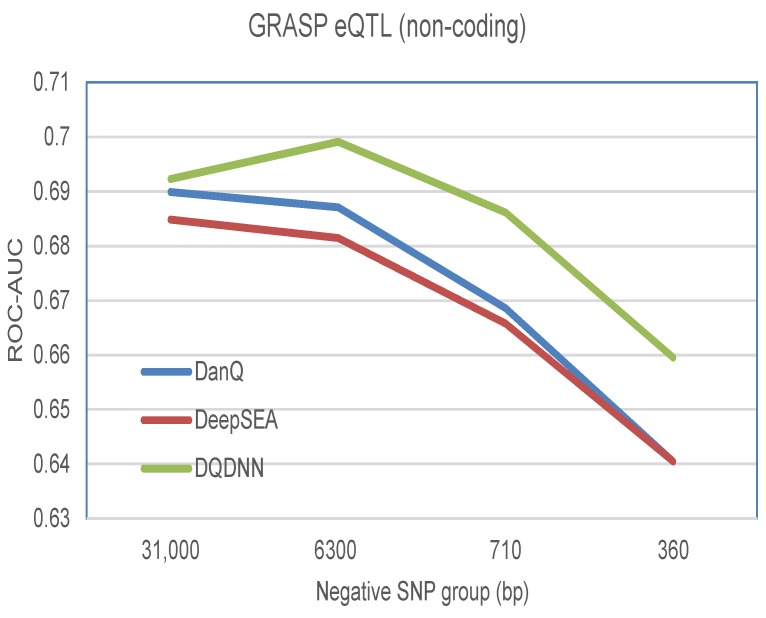
Comparison of the the proposed model and the DanQ and DeepSEA models for prioritizing functionally annotated genome-wide repository of associations between SNPs and phenotypes (GRASP) quantitative trait loci (eQTLs) SNPs against 1000 Genomes Project noncoding SNPs across several negative SNP groups of varying distances to the positive SNPs.

The performance of the MLP model was estimated by 10-fold cross-validation and across the several negative groups, as shown in Table 7. Figure 12 shows that the proposed model outperformed the DanQ and DeepSEA models in all negative groups.

## 4. Conclusions

The understanding of non-coding regions in DNA is an important step, as many of the disease associated variants are located in these regions. Therefore, we introduced a deep learning model for quantifying these regions into 919 targets. We showed that the evolutionary information helped to improve the classification performance. Furthermore, we designed a well optimized deep learning model that outperformed the state-of-the-art-models in terms of ROC-AUC and PR-AUC. Multi-scale motif learning helped with capturing motifs at different lengths, and the recurrent neural networks helped with studying the relations between the discovered motifs in the first layers. In addition, we showed that the proposed model could be used for functional SNP prioritization and outperformed the comparative methods. All trained models and weights have been made available at https://home.jbnu.ac.kr/NSCL/data/DQDNN/DQDNN.zip.

## Figures and Tables

**Table 1:** The detailed parameters used in the forward/reverse complement network.

Layer	Output Shape
Input	(1000,5)
ine Conv1D(256,7,1)	(1000,256)
Conv1D(256,13,1)	(1000,256)
Conv1D(256,26,1)	(1000,256)
ine Concatenate	(1000,768)
ine Max_pooling_1D(7,7)	(142,768)
Dropout(0.4)	(142,768)
BiLSTM(256)	(142,512)
BiLSTM(256)	(142,512)
Max_pooling_1D(13,13)	(10,512)
Dropout(0.5)	(10,512)
Flatten()	5120

**Table 2:** The detailed parameters used in the MLP classifier network.

Layer	Output Shape
Input	5120
Dense(512)	512
ReLU	512
Dense(919)	919
Sigmoid	919

**Table 3:** The configurations of the MLP model for functional SNP prioritization.

Layer	Output Shape
Input	3676
Dropout(0.3)	3676
Dense(256)	256
ReLU	256
Dropout(0.5)	256
Dropout(1)	1
Sigmoid	1

**Table 4:** Performance comparison between using raw DNA sequences only and by integrating conservation scores (CONS) with the raw DNA sequences. PR, precision-recall.

	ROC-AUC		PR-AUC	
	**DQDNN-DNA**	**DQDNN-CONS**	**DQDNN-DNA**	**DQDNN-CONS**
DNase I	0.9190	0.9223	0.4779	0.4986
TF	0.9580	0.9612	0.3740	0.3905
Histone marks	0.8619	0.8827	0.3896	0.4297
ALL	0.9428	0.9480	0.3905	0.4102

**Table 5:** Performance comparison in terms of the average ROC-AUC between the proposed model and the DanQand DeepSEA models.

	DeepSEA	DanQ	DQDNN-DNA	DQDNN-CONS
DNase I	0.9082	0.9173	0.9190	0.9223
TF	0.9478	0.9568	0.9580	0.9612
Histone marks	0.8522	0.8621	0.8619	0.8827
ALL	0.9325	0.9417	0.9428	0.9480

**Table 6:** Performance comparison in terms of the average PR-AUC between the proposed model and the DanQ and DeepSEA models.

	DeepSEA	DanQ	DQDNN-DNA	DQDNN-CONS
DNase I	0.4407	0.4714	0.4779	0.4986
TF	0.3203	0.3606	0.3740	0.3905
Histone marks	0.3676	0.3882	0.3896	0.4297
ALL	0.3425	0.3794	0.3905	0.4102

**Table 7:** The of ROC-AUC of 10-fold cross-validation of the functional SNP prioritization model.

	Negative SNP Group (bp)			
**Folds**	**31,000 bp**	**6300 bp**	**710 bp**	**360 bp**
Fold 0	0.7048	0.7154	0.6981	0.6752
Fold 1	0.6763	0.6799	0.6877	0.6605
Fold 2	0.6948	0.7072	0.7002	0.6580
Fold 3	0.7032	0.7198	0.7083	0.6737
Fold 4	0.7105	0.7049	0.6900	0.6625
Fold 5	0.7221	0.7111	0.6985	0.6756
Fold 6	0.6772	0.6922	0.6623	0.6490
Fold 7	0.6611	0.6745	0.6657	0.6308
Fold 8	0.6888	0.6927	0.6727	0.6457
Fold 9	0.6840	0.6933	0.6778	0.6642
ine **Average**	0.6923	0.6991	0.6861	0.6595
ine **STD Error**	0.0184	0.0150	0.0158	0.0144

## Supplementary Materials

Supplementary materials can be found at https://www.mdpi.com/2073-4409/8/12/1635/s1. Supplementary File 1: The peroformance comparison of DQDNN model and DeepSEA and DanQ in terms of ROC-AUC and PR-AUC.
