# Supplementary material for: Improving the Quantification of DNA Sequences Using Evolutionary Information Based on Deep Learning
Source: Cells. 2019 Dec 14;8(12):1635. doi: 10.3390/cells8121635 (PMC6952993; doi:10.3390/cells8121635)
Supplement: Supplementary file 1 [file cells-08-01635-s001.pdf]

The peroformance comparison of DQDNN model and DeepSEA and DanQ in terms of ROC-AUC and PR-AUC

idx	Cell Type	TF/DNase/HistoneMark	DQDNN-CONS PR-AUC	DQDNN-CONS ROC-AUC	DQDNN-DNA PR-AUC	DQDNN-DNA ROC-AUC	DeepSEA ROC AUC	DanQ ROC AUC	DeepSEA PR AUC	DanQ PR AUC
0	8988T	DNase	0.4368	0.9185	0.4261	0.9157	0.9104	0.9132	0.4011	0.4184
1	AoSMC	DNase	0.5033	0.9379	0.4744	0.9346	0.9245	0.9278	0.4396	0.4572
2	Chorion	DNase	0.3518	0.9058	0.3412	0.9029	0.8963	0.9003	0.3207	0.3385
3	CLL	DNase	0.3958	0.9345	0.3673	0.9311	0.9283	0.9303	0.3592	0.3704
4	Fibrobl	DNase	0.3763	0.8494	0.3619	0.8446	0.8379	0.8407	0.3407	0.3526
5	FibroP	DNase	0.4695	0.8975	0.4498	0.8934	0.8833	0.8873	0.4208	0.4352
6	Gliobla	DNase	0.4840	0.9237	0.4723	0.9211	0.9124	0.9166	0.4430	0.4603
7	GM12891	DNase	0.4598	0.9380	0.4454	0.9346	0.9297	0.9335	0.4206	0.4421
8	GM12892	DNase	0.4287	0.9315	0.4187	0.9290	0.9243	0.9272	0.3935	0.4125
9	GM18507	DNase	0.4685	0.9379	0.4467	0.9356	0.9276	0.9311	0.4168	0.4392
10	GM19238	DNase	0.4530	0.9293	0.4376	0.9266	0.9188	0.9220	0.4102	0.4302
11	GM19239	DNase	0.4504	0.9359	0.4353	0.9329	0.9264	0.9295	0.4100	0.4274
12	GM19240	DNase	0.4259	0.9105	0.4100	0.9067	0.8997	0.9028	0.3874	0.4021
13	H9ES	DNase	0.5367	0.9488	0.5332	0.9483	0.9392	0.9457	0.4914	0.5213
14	HeLa-S3	DNase	0.4531	0.9227	0.4308	0.9193	0.9061	0.9136	0.3955	0.4161
15	Hepatocytes	DNase	0.3201	0.8923	0.3029	0.8865	0.8820	0.8856	0.2856	0.3027
16	HPDE6-E6E7	DNase	0.4890	0.9441	0.4691	0.9414	0.9283	0.9368	0.4250	0.4522
17	HSMM_emb	DNase	0.4946	0.9444	0.4696	0.9412	0.9329	0.9371	0.4443	0.4616
18	HTR8svn	DNase	0.4734	0.9379	0.4515	0.9343	0.9248	0.9295	0.4174	0.4367
19	Huh-7.5	DNase	0.4440	0.9163	0.4278	0.9121	0.9019	0.9077	0.3974	0.4177

20	Huh-7	DNase	0.4664	0.9298	0.4521	0.9267	0.9172	0.9225	0.4167	0.4414
21	iPS	DNase	0.5389	0.9506	0.5345	0.9501	0.9447	0.9486	0.5074	0.5310
22	Ishikawa	DNase	0.4678	0.9204	0.4554	0.9178	0.9064	0.9157	0.4170	0.4480
23	Ishikawa	DNase	0.4734	0.9220	0.4584	0.9185	0.9082	0.9162	0.4229	0.4496
24	LNCaP	DNase	0.4759	0.9322	0.4597	0.9301	0.9179	0.9272	0.4195	0.4512
25	MCF-7	DNase	0.4497	0.9164	0.4375	0.9141	0.9025	0.9121	0.4006	0.4282
26	Medullo	DNase	0.3412	0.8705	0.3328	0.8671	0.8640	0.8664	0.3158	0.3272
27	Melano	DNase	0.3935	0.8598	0.3741	0.8550	0.8474	0.8507	0.3536	0.3663
28	Myometr	DNase	0.4725	0.9291	0.4544	0.9260	0.9160	0.9213	0.4186	0.4397
29	Osteobl	DNase	0.3847	0.8610	0.3704	0.8573	0.8493	0.8538	0.3504	0.3636
30	PanIsletD	DNase	0.5111	0.9265	0.4913	0.9233	0.9141	0.9188	0.4576	0.4791
31	PanIslets	DNase	0.4082	0.9016	0.3920	0.8972	0.8922	0.8966	0.3708	0.3899
32	pHTE	DNase	0.4668	0.9056	0.4571	0.9032	0.8925	0.9001	0.4236	0.4501
33	ProgFib	DNase	0.4962	0.9267	0.4752	0.9234	0.9153	0.9191	0.4406	0.4626
34	RWPE1	DNase	0.5403	0.9433	0.5229	0.9407	0.9277	0.9366	0.4744	0.5071
35	Stellate	DNase	0.5121	0.9404	0.4854	0.9358	0.9258	0.9312	0.4496	0.4722
36	T-47D	DNase	0.4164	0.9041	0.4003	0.9011	0.8895	0.8982	0.3707	0.3961
37	Adult_CD4_Th0	DNase	0.4012	0.8943	0.3852	0.8896	0.8848	0.8887	0.3712	0.3825
38	Urothelia	DNase	0.5017	0.9350	0.4867	0.9322	0.9219	0.9283	0.4499	0.4732
39	Urothelia	DNase	0.4707	0.9164	0.4578	0.9139	0.9052	0.9110	0.4219	0.4431
40	AG04449	DNase	0.5408	0.9470	0.5051	0.9427	0.9303	0.9351	0.4583	0.4757

41	AG04450	DNase	0.5345	0.9469	0.5000	0.9426	0.9306	0.9344	0.4541	0.4672
42	AG09309	DNase	0.5788	0.9471	0.5432	0.9430	0.9295	0.9340	0.4960	0.5116
43	AG09319	DNase	0.5211	0.9459	0.4819	0.9399	0.9278	0.9316	0.4357	0.4508
44	AG10803	DNase	0.5727	0.9510	0.5369	0.9467	0.9351	0.9386	0.4908	0.5055
45	AoAF	DNase	0.5589	0.9454	0.5237	0.9404	0.9281	0.9315	0.4824	0.4957
46	BE2_C	DNase	0.5185	0.9294	0.5053	0.9283	0.9132	0.9234	0.4558	0.4877
47	BJ	DNase	0.5247	0.9438	0.4872	0.9391	0.9260	0.9300	0.4400	0.4584
48	Caco-2	DNase	0.4752	0.9618	0.4750	0.9629	0.9551	0.9602	0.4414	0.4660
49	CD20+	DNase	0.4351	0.9291	0.4097	0.9241	0.9174	0.9213	0.3937	0.4087
50	CD34+_Mobilized	DNase	0.5536	0.9405	0.5328	0.9379	0.9255	0.9316	0.4920	0.5126
51	CMK	DNase	0.4754	0.9399	0.4583	0.9366	0.9287	0.9327	0.4316	0.4520
52	A549	DNase	0.4958	0.9071	0.4815	0.9032	0.8891	0.8962	0.4455	0.4649
53	GM12878	DNase	0.4517	0.9097	0.4292	0.9037	0.8953	0.9001	0.4034	0.4227
54	H1-hESC	DNase	0.5403	0.9329	0.5359	0.9325	0.9229	0.9282	0.4978	0.5223
55	HeLa-S3	DNase	0.4680	0.8992	0.4526	0.8955	0.8814	0.8896	0.4132	0.4332
56	HepG2	DNase	0.5174	0.9334	0.5082	0.9322	0.9196	0.9243	0.4674	0.4850
57	HMEC	DNase	0.4926	0.8932	0.4834	0.8912	0.8793	0.8867	0.4488	0.4683
58	HSMMtube	DNase	0.5751	0.9256	0.5551	0.9231	0.9117	0.9151	0.5158	0.5322
59	HSMM	DNase	0.5535	0.9235	0.5301	0.9205	0.9070	0.9119	0.4915	0.5070
60	HUVEC	DNase	0.4958	0.9211	0.4754	0.9166	0.9057	0.9103	0.4434	0.4584
61	K562	DNase	0.4811	0.9184	0.4614	0.9141	0.9035	0.9095	0.4312	0.4488
62	LNCaP	DNase	0.4722	0.8955	0.4636	0.8936	0.8758	0.8880	0.4156	0.4474
63	MCF-7	DNase	0.4776	0.9106	0.4658	0.9088	0.8877	0.9003	0.4127	0.4463
64	NHEK	DNase	0.5300	0.9308	0.5112	0.9266	0.9141	0.9229	0.4671	0.4960

65	Th1	DNase	0.3862	0.8748	0.3756	0.8695	0.8652	0.8685	0.3583	0.3697
66	GM06990	DNase	0.3690	0.9151	0.3544	0.9122	0.9030	0.9063	0.3257	0.3396
67	GM12864	DNase	0.4739	0.9295	0.4514	0.9268	0.9179	0.9204	0.4163	0.4328
68	GM12865	DNase	0.5160	0.9380	0.4976	0.9357	0.9263	0.9290	0.4562	0.4756
69	H7-hESC	DNase	0.5526	0.9298	0.5430	0.9285	0.9155	0.9227	0.4913	0.5167
70	HAc	DNase	0.5608	0.9441	0.5338	0.9404	0.9285	0.9328	0.4888	0.5050
71	HAEpiC	DNase	0.5564	0.9404	0.5303	0.9370	0.9266	0.9298	0.4905	0.5048
72	HA-h	DNase	0.5549	0.9345	0.5309	0.9312	0.9187	0.9240	0.4878	0.5082
73	HA-sp	DNase	0.3477	0.8877	0.3324	0.8810	0.8676	0.8750	0.3104	0.3207
74	HBMEC	DNase	0.5803	0.9423	0.5569	0.9395	0.9276	0.9320	0.5143	0.5323
75	HCFaa	DNase	0.5697	0.9431	0.5416	0.9397	0.9259	0.9315	0.4933	0.5149
76	HCF	DNase	0.5717	0.9470	0.5401	0.9435	0.9313	0.9350	0.4958	0.5093
77	HCM	DNase	0.5935	0.9478	0.5663	0.9454	0.9328	0.9371	0.5207	0.5356
78	HConF	DNase	0.5649	0.9462	0.5320	0.9419	0.9294	0.9347	0.4829	0.4997
79	HCPEpiC	DNase	0.5732	0.9389	0.5456	0.9351	0.9201	0.9258	0.4972	0.5178
80	HCT-116	DNase	0.5512	0.9410	0.5265	0.9376	0.9251	0.9336	0.4877	0.5088
81	HEEpiC	DNase	0.6277	0.9520	0.6145	0.9504	0.9341	0.9448	0.5501	0.5846
82	HFF-Myc	DNase	0.5511	0.9324	0.5297	0.9297	0.9143	0.9202	0.4832	0.4994
83	HFF	DNase	0.5801	0.9424	0.5490	0.9388	0.9251	0.9302	0.5030	0.5197
84	HGF	DNase	0.5133	0.9439	0.4797	0.9399	0.9279	0.9318	0.4366	0.4493
85	HIPEpiC	DNase	0.5850	0.9416	0.5569	0.9383	0.9246	0.9294	0.5103	0.5272
86	HL-60	DNase	0.4634	0.9206	0.4480	0.9170	0.9092	0.9129	0.4205	0.4341
87	HMF	DNase	0.6043	0.9518	0.5764	0.9487	0.9365	0.9413	0.5294	0.5450
88	HMVEC-dAd	DNase	0.5185	0.9435	0.4864	0.9387	0.9273	0.9327	0.4481	0.4614
89	HMVEC-dBl-Ad	DNase	0.5678	0.9498	0.5367	0.9456	0.9339	0.9389	0.4886	0.5059

90	HMVEC-dBl-Neo	DNase	0.5571	0.9422	0.5310	0.9377	0.9249	0.9306	0.4836	0.5032
91	HMVEC-dLy-Ad	DNase	0.5076	0.9399	0.4809	0.9361	0.9275	0.9314	0.4488	0.4643
92	HMVEC-dLy-Neo	DNase	0.5569	0.9465	0.5276	0.9429	0.9308	0.9356	0.4794	0.4969
93	HMVEC-dNeo	DNase	0.5490	0.9451	0.5217	0.9420	0.9300	0.9350	0.4779	0.4938
94	HMVEC-LBl	DNase	0.5743	0.9465	0.5392	0.9421	0.9303	0.9356	0.4962	0.5136
95	HMVEC-LLy	DNase	0.5223	0.9376	0.4984	0.9333	0.9237	0.9284	0.4642	0.4804
96	HNPCEpiC	DNase	0.6047	0.9470	0.5814	0.9439	0.9299	0.9343	0.5292	0.5480
97	HPAEC	DNase	0.5049	0.9415	0.4739	0.9377	0.9249	0.9303	0.4289	0.4472
98	HPAF	DNase	0.6040	0.9495	0.5749	0.9462	0.9335	0.9385	0.5276	0.5444
99	HPdLF	DNase	0.5398	0.9440	0.5000	0.9386	0.9264	0.9305	0.4581	0.4726
100	HPF	DNase	0.5574	0.9470	0.5231	0.9432	0.9304	0.9351	0.4797	0.4970
101	HRCEpiC	DNase	0.5481	0.9376	0.5320	0.9358	0.9213	0.9259	0.4844	0.4974
102	HRE	DNase	0.5615	0.9404	0.5405	0.9370	0.9236	0.9279	0.4948	0.5102
103	HRGEC	DNase	0.4877	0.9293	0.4641	0.9248	0.9101	0.9163	0.4216	0.4378
104	HRPEpiC	DNase	0.5207	0.9283	0.5028	0.9261	0.9136	0.9191	0.4596	0.4778
105	HVMF	DNase	0.4866	0.9280	0.4522	0.9230	0.9111	0.9153	0.4235	0.4342
106	Jurkat	DNase	0.5112	0.9339	0.4991	0.9320	0.9215	0.9284	0.4620	0.4887
107	Monocytes-CD14+_RO01746	DNase	0.5166	0.9468	0.4874	0.9426	0.9367	0.9406	0.4595	0.4781
108	NB4	DNase	0.5279	0.9455	0.5025	0.9427	0.9311	0.9378	0.4632	0.4887

109	NH-A	DNase	0.5644	0.9464	0.5384	0.9427	0.9315	0.9348	0.4957	0.5083
110	NHDF-Ad	DNase	0.5750	0.9444	0.5515	0.9414	0.9307	0.9333	0.5077	0.5215
111	NHDF-neo	DNase	0.5339	0.9414	0.5019	0.9371	0.9257	0.9281	0.4580	0.4691
112	NHLF	DNase	0.5835	0.9480	0.5587	0.9449	0.9335	0.9374	0.5139	0.5302
113	NT2-D1	DNase	0.5636	0.9411	0.5559	0.9404	0.9271	0.9340	0.5022	0.5325
114	PANC-1	DNase	0.4764	0.9240	0.4583	0.9207	0.9081	0.9153	0.4218	0.4435
115	PrEC	DNase	0.5615	0.9475	0.5462	0.9456	0.9318	0.9409	0.4915	0.5208
116	RPTEC	DNase	0.5007	0.9292	0.4842	0.9265	0.9115	0.9164	0.4439	0.4598
117	SAEC	DNase	0.6225	0.9523	0.6050	0.9514	0.9349	0.9449	0.5430	0.5760
118	SKMC	DNase	0.5668	0.9423	0.5391	0.9394	0.9263	0.9315	0.4925	0.5093
119	SK-N-MC	DNase	0.4283	0.9021	0.4150	0.8996	0.8830	0.8943	0.3707	0.4057
120	SK-N-SH_RA	DNase	0.4531	0.9481	0.4372	0.9466	0.9391	0.9439	0.3975	0.4262
121	Th2	DNase	0.3660	0.9172	0.3486	0.9124	0.9050	0.9104	0.3294	0.3493
122	WERI-Rb-1	DNase	0.4689	0.9223	0.4573	0.9217	0.9136	0.9173	0.4279	0.4437
123	WI-38	DNase	0.5085	0.9268	0.4781	0.9233	0.9131	0.9156	0.4480	0.4590
124	WI-38	DNase	0.5437	0.9444	0.5054	0.9406	0.9276	0.9327	0.4625	0.4808
125	Dnd41	CTCF	0.7383	0.9840	0.7339	0.9833	0.9792	0.9828	0.6948	0.7280
126	Dnd41	EZH2	0.0530	0.9582	0.0484	0.9547	0.9449	0.9505	0.0504	0.0530
127	GM12878	CTCF	0.7308	0.9848	0.7140	0.9840	0.9784	0.9820	0.6663	0.7017
128	GM12878	EZH2	0.0544	0.9487	0.0549	0.9359	0.9194	0.9337	0.0469	0.0550
129	H1-hESC	CHD1	0.2186	0.9568	0.2019	0.9521	0.9472	0.9523	0.1904	0.1998
130	H1-hESC	CTCF	0.7005	0.9847	0.7014	0.9842	0.9774	0.9820	0.6404	0.6851
131	H1-hESC	EZH2	0.5281	0.9871	0.4584	0.9841	0.9817	0.9843	0.3951	0.4475

132	H1-hESC	JARID1A	0.3020	0.9894	0.2802	0.9850	0.9872	0.9836	0.1856	0.2526
133	H1-hESC	RBBP5	0.4557	0.9716	0.4369	0.9718	0.9650	0.9690	0.4278	0.4431
134	HeLa-S3	CTCF	0.7055	0.9754	0.7015	0.9748	0.9650	0.9711	0.6521	0.6900
135	HeLa-S3	EZH2	0.0383	0.8608	0.0310	0.8507	0.8101	0.8440	0.0187	0.0294
136	HeLa-S3	Pol2(b)	0.1521	0.9154	0.1212	0.8972	0.8907	0.9011	0.1088	0.1127
137	HepG2	CTCF	0.7338	0.9889	0.7206	0.9884	0.9835	0.9865	0.6687	0.7080
138	HepG2	EZH2	0.0884	0.9604	0.0651	0.9547	0.9517	0.9567	0.0610	0.0651
139	HMEC	CTCF	0.7281	0.9894	0.7158	0.9888	0.9846	0.9876	0.6617	0.6979
140	HMEC	EZH2	0.2445	0.9699	0.2208	0.9659	0.9593	0.9640	0.2024	0.2178
141	HSMM	CTCF	0.6884	0.9842	0.6783	0.9835	0.9762	0.9809	0.6278	0.6645
142	HSMM	EZH2	0.1294	0.9779	0.1204	0.9774	0.9612	0.9695	0.1066	0.1145
143	HSMMtube	CTCF	0.6960	0.9806	0.6899	0.9807	0.9726	0.9779	0.6378	0.6742
144	HSMMtube	EZH2	0.2292	0.9872	0.2067	0.9854	0.9842	0.9854	0.1645	0.1947
145	HUVEC	CTCF	0.7250	0.9877	0.7098	0.9864	0.9822	0.9848	0.6652	0.6971
146	HUVEC	EZH2	0.4576	0.9887	0.4174	0.9874	0.9856	0.9872	0.3573	0.4005
147	HUVEC	Pol2(b)	0.2164	0.9450	0.1865	0.9357	0.9240	0.9315	0.1687	0.1811
148	K562	CHD1	0.2406	0.9619	0.2192	0.9590	0.9548	0.9590	0.2158	0.2292
149	K562	CTCF	0.6853	0.9794	0.6747	0.9785	0.9705	0.9758	0.6238	0.6608
150	K562	EZH2	0.0298	0.9183	0.0344	0.9316	0.8878	0.9233	0.0345	0.0521
151	K562	HDAC1	0.3760	0.9694	0.3497	0.9656	0.9655	0.9678	0.3205	0.3432
152	K562	HDAC2	0.1414	0.9642	0.1299	0.9611	0.9522	0.9543	0.1036	0.1159
153	K562	HDAC6	0.0707	0.9825	0.0397	0.9754	0.9801	0.9821	0.0149	0.0535
154	K562	p300	0.1796	0.9243	0.2028	0.9207	0.9054	0.9142	0.0952	0.1862
155	K562	PHF8	0.7402	0.9863	0.7164	0.9860	0.9836	0.9861	0.6930	0.7236
156	K562	PLU1	0.5010	0.9712	0.4712	0.9681	0.9650	0.9693	0.4451	0.4784
157	K562	Pol2(b)	0.3753	0.9569	0.3374	0.9521	0.9457	0.9522	0.2919	0.3261
158	K562	RBBP5	0.4958	0.9685	0.4606	0.9655	0.9612	0.9645	0.4377	0.4645

159	K562	SAP30	0.4428	0.9872	0.4114	0.9859	0.9839	0.9852	0.3695	0.3931
160	NH-A	CTCF	0.7204	0.9871	0.7034	0.9865	0.9803	0.9846	0.6581	0.6876
161	NH-A	EZH2	0.4537	0.9895	0.4010	0.9879	0.9867	0.9884	0.3599	0.3987
162	NHDF-Ad	CTCF	0.6821	0.9841	0.6632	0.9831	0.9761	0.9804	0.6185	0.6535
163	NHDF-Ad	EZH2	0.3750	0.9888	0.3500	0.9869	0.9857	0.9876	0.3151	0.3338
164	NHEK	CTCF	0.7007	0.9820	0.6821	0.9807	0.9728	0.9778	0.6343	0.6671
165	NHEK	EZH2	0.3521	0.9820	0.3411	0.9813	0.9785	0.9800	0.3021	0.3157
166	NHEK	Pol2(b)	0.1315	0.9039	0.0960	0.8744	0.8580	0.8703	0.0887	0.0983
167	NHLF	CTCF	0.7081	0.9872	0.6776	0.9860	0.9805	0.9839	0.6336	0.6708
168	NHLF	EZH2	0.3081	0.9791	0.2862	0.9788	0.9747	0.9776	0.2605	0.2815
169	Osteobl	CTCF	0.7294	0.9767	0.7175	0.9765	0.9653	0.9720	0.6702	0.7028
170	A549	ATF3	0.2144	0.9431	0.1749	0.9311	0.9132	0.9264	0.1157	0.1526
171	A549	BCL3	0.1620	0.9005	0.1369	0.8889	0.8677	0.8856	0.0966	0.1252
172	A549	CREB1	0.4892	0.9736	0.4706	0.9712	0.9657	0.9707	0.4403	0.4606
173	A549	CTCF	0.6231	0.9905	0.6068	0.9900	0.9878	0.9890	0.5616	0.5953
174	A549	CTCF	0.6559	0.9907	0.6463	0.9898	0.9871	0.9890	0.6045	0.6367
175	A549	ELF1	0.3647	0.9546	0.3395	0.9490	0.9381	0.9430	0.2848	0.3125
176	A549	ETS1	0.3745	0.9592	0.3498	0.9555	0.9435	0.9540	0.2728	0.3287
177	A549	FOSL2	0.4047	0.9491	0.3839	0.9453	0.9327	0.9389	0.3437	0.3584
178	A549	FOXA1	0.0730	0.9197	0.0700	0.9189	0.9009	0.9088	0.0546	0.0667
179	A549	GABP	0.4645	0.9589	0.4349	0.9530	0.9430	0.9508	0.3658	0.4093
180	A549	GR	0.0690	0.9524	0.0862	0.9360	0.9228	0.9298	0.0266	0.0499
181	A549	GR	0.1750	0.8778	0.1585	0.8761	0.8636	0.8794	0.1399	0.1568
182	A549	GR	0.1590	0.9137	0.1307	0.9053	0.8915	0.9035	0.1127	0.1257
183	A549	GR	0.1830	0.9027	0.1716	0.8993	0.8893	0.9071	0.1504	0.1696
184	A549	NRSF	0.3819	0.9418	0.3569	0.9352	0.9048	0.9124	0.2423	0.2976
185	A549	p300	0.2591	0.9136	0.2187	0.9038	0.8834	0.8945	0.1796	0.2020
186	A549	Pol2	0.6456	0.9639	0.6310	0.9618	0.9592	0.9620	0.6167	0.6291
187	A549	Pol2	0.6695	0.9654	0.6531	0.9635	0.9605	0.9628	0.6341	0.6475
188	A549	Sin3Ak-20	0.1493	0.9117	0.1337	0.9034	0.8854	0.8994	0.1022	0.1214
189	A549	SIX5	0.3723	0.9590	0.3486	0.9542	0.9406	0.9518	0.2472	0.3205

190	A549	TAF1	0.4324	0.9516	0.4044	0.9448	0.9340	0.9405	0.3616	0.3900
191	A549	TCF12	0.2832	0.9142	0.2538	0.9056	0.8819	0.8955	0.2044	0.2329
192	A549	USF1	0.3288	0.9645	0.3080	0.9589	0.9451	0.9537	0.2417	0.2813
193	A549	USF1	0.4254	0.9819	0.4123	0.9800	0.9683	0.9729	0.3307	0.3800
194	A549	USF1	0.3498	0.9612	0.3378	0.9573	0.9305	0.9470	0.2454	0.3077
195	A549	YY1	0.4040	0.9615	0.3763	0.9561	0.9413	0.9494	0.3179	0.3350
196	A549	ZBTB33	0.2564	0.9435	0.2343	0.9328	0.9131	0.9266	0.1468	0.1915
197	ECC-1	CTCF	0.8060	0.9959	0.7861	0.9961	0.9949	0.9958	0.7529	0.7824
198	ECC-1	ERalpha	0.0522	0.8997	0.0427	0.8990	0.8789	0.8897	0.0273	0.0379
199	ECC-1	ERalpha	0.1022	0.8705	0.0971	0.8713	0.8541	0.8611	0.0741	0.0863
200	ECC-1	ERalpha	0.1107	0.8881	0.0975	0.8847	0.8639	0.8739	0.0647	0.0863
201	ECC-1	FOXA1	0.1124	0.9491	0.1061	0.9404	0.9209	0.9319	0.0698	0.0885
202	ECC-1	GR	0.0945	0.9032	0.0790	0.8966	0.8752	0.8964	0.0588	0.0700
203	ECC-1	Pol2	0.6513	0.9754	0.6206	0.9723	0.9706	0.9726	0.5920	0.6155
204	GM12878	ATF2	0.3028	0.9295	0.2685	0.9243	0.9082	0.9133	0.2242	0.2422
205	GM12878	ATF3	0.3582	0.9922	0.3552	0.9922	0.9719	0.9867	0.2339	0.3314
206	GM12878	BATF	0.3249	0.9499	0.2973	0.9470	0.9320	0.9431	0.2294	0.2767
207	GM12878	BCL11A	0.2511	0.9543	0.2183	0.9506	0.9427	0.9468	0.1762	0.1935
208	GM12878	BCL3	0.2156	0.9173	0.1942	0.9105	0.9046	0.9108	0.1674	0.1900
209	GM12878	BCLAF1	0.2073	0.9664	0.1782	0.9590	0.9584	0.9581	0.1373	0.1725
210	GM12878	CEBPB	0.1815	0.9346	0.1507	0.9303	0.9170	0.9226	0.1024	0.1288
211	GM12878	EBF1	0.2897	0.9369	0.2618	0.9336	0.9040	0.9302	0.1809	0.2410
212	GM12878	Egr-1	0.3363	0.9764	0.3207	0.9765	0.9681	0.9702	0.2555	0.2900
213	GM12878	ELF1	0.5583	0.9782	0.5282	0.9763	0.9717	0.9750	0.4803	0.5151

214	GM12878	ETS1	0.3430	0.9872	0.3542	0.9865	0.9856	0.9834	0.2599	0.3159
215	GM12878	FOXM1	0.2726	0.9290	0.2413	0.9233	0.9080	0.9147	0.2021	0.2239
216	GM12878	GABP	0.6277	0.9905	0.6016	0.9898	0.9876	0.9888	0.5212	0.5880
217	GM12878	IRF4	0.2631	0.9517	0.2348	0.9493	0.9345	0.9449	0.1759	0.2088
218	GM12878	MEF2A	0.2475	0.9503	0.2145	0.9451	0.9293	0.9277	0.1542	0.1610
219	GM12878	MEF2C	0.2060	0.9567	0.1546	0.9515	0.9351	0.9279	0.0957	0.1017
220	GM12878	MTA3	0.2117	0.9187	0.1813	0.9095	0.8999	0.9058	0.1468	0.1674
221	GM12878	NFATC1	0.2195	0.9160	0.2071	0.9094	0.8970	0.9030	0.1669	0.1920
222	GM12878	NFIC	0.3277	0.9190	0.2924	0.9148	0.9001	0.9050	0.2451	0.2655
223	GM12878	NRSF	0.5498	0.9793	0.5371	0.9826	0.9728	0.9566	0.4368	0.4477
224	GM12878	p300	0.1940	0.9704	0.1562	0.9614	0.9580	0.9594	0.1115	0.1289
225	GM12878	PAX5-C20	0.2213	0.9135	0.1976	0.9098	0.9007	0.9062	0.1648	0.1879
226	GM12878	PAX5-N19	0.2277	0.9263	0.2034	0.9216	0.9153	0.9202	0.1698	0.1733
227	GM12878	Pbx3	0.1307	0.9385	0.1268	0.9399	0.9315	0.9336	0.1119	0.1191
228	GM12878	PML	0.4080	0.9457	0.3655	0.9361	0.9269	0.9329	0.3132	0.3493
229	GM12878	Pol2-4H8	0.4258	0.9084	0.3866	0.8962	0.8929	0.8991	0.3619	0.3853
230	GM12878	Pol2	0.6362	0.9618	0.6099	0.9589	0.9574	0.9598	0.5922	0.6094

231	GM12878	POU2F2	0.2874	0.9483	0.2570	0.9415	0.9257	0.9231	0.1981	0.2117
232	GM12878	PU.1	0.5388	0.9751	0.5221	0.9733	0.9632	0.9632	0.4392	0.4560
233	GM12878	Rad21	0.6274	0.9849	0.6129	0.9842	0.9801	0.9828	0.5819	0.6097
234	GM12878	RUNX3	0.4569	0.9304	0.4212	0.9274	0.9189	0.9218	0.3849	0.3970
235	GM12878	RXRA	0.0592	0.9832	0.0506	0.9776	0.9755	0.9725	0.0232	0.0565
236	GM12878	SIX5	0.6237	0.9918	0.6149	0.9910	0.9907	0.9928	0.4758	0.6106
237	GM12878	SP1	0.4003	0.9666	0.3712	0.9617	0.9551	0.9578	0.3273	0.3505
238	GM12878	SRF	0.1877	0.9351	0.1734	0.9341	0.9265	0.9294	0.1406	0.1697
239	GM12878	STAT5A	0.1521	0.9198	0.1319	0.9107	0.9005	0.9075	0.1029	0.1254
240	GM12878	TAF1	0.4276	0.9703	0.3978	0.9652	0.9621	0.9638	0.3601	0.3909
241	GM12878	TCF12	0.2782	0.9600	0.2559	0.9595	0.9487	0.9549	0.2004	0.2299
242	GM12878	TCF3	0.2631	0.9582	0.2366	0.9582	0.9480	0.9537	0.1802	0.2149
243	GM12878	USF1	0.4344	0.9868	0.4228	0.9860	0.9825	0.9836	0.3840	0.3952
244	GM12878	YY1	0.4763	0.9608	0.4504	0.9598	0.9520	0.9569	0.4038	0.4407
245	GM12878	ZBTB33	0.1277	0.9797	0.2238	0.9775	0.9695	0.9765	0.0673	0.1074
246	GM12878	ZEB1	0.1978	0.9742	0.1762	0.9705	0.9668	0.9717	0.1701	0.1904
247	GM12891	PAX5-C20	0.0398	0.9271	0.0300	0.9229	0.9172	0.9244	0.0262	0.0323

248	GM12891	Pol2-4H8	0.3554	0.9153	0.3287	0.9035	0.9009	0.9066	0.3019	0.3173
249	GM12891	Pol2	0.3896	0.9217	0.3572	0.9153	0.9090	0.9152	0.3311	0.3467
250	GM12891	POU2F2	0.2296	0.9467	0.2070	0.9467	0.9305	0.9286	0.1550	0.1744
251	GM12891	PU.1	0.5468	0.9740	0.5298	0.9726	0.9631	0.9640	0.4392	0.4623
252	GM12891	TAF1	0.4265	0.9695	0.4083	0.9660	0.9639	0.9654	0.3581	0.3884
253	GM12891	YY1	0.4414	0.9746	0.4208	0.9721	0.9667	0.9713	0.3703	0.4097
254	GM12892	PAX5-C20	0.3102	0.9588	0.2908	0.9561	0.9517	0.9547	0.2678	0.2806
255	GM12892	Pol2-4H8	0.3698	0.9150	0.3410	0.9041	0.8992	0.9069	0.3082	0.3342
256	GM12892	Pol2	0.5122	0.9384	0.4771	0.9352	0.9309	0.9353	0.4494	0.4784
257	GM12892	TAF1	0.4479	0.9758	0.4200	0.9747	0.9707	0.9738	0.3889	0.4188
258	GM12892	YY1	0.4242	0.9670	0.3968	0.9628	0.9546	0.9620	0.3430	0.3750
259	H1-hESC	ATF2	0.1790	0.9435	0.1791	0.9408	0.9148	0.9368	0.1143	0.1460
260	H1-hESC	ATF3	0.4001	0.9885	0.3805	0.9885	0.9825	0.9870	0.2733	0.3494
261	H1-hESC	BCL11A	0.1497	0.9614	0.1692	0.9597	0.9400	0.9562	0.0674	0.1063
262	H1-hESC	CTCF	0.6522	0.9883	0.6446	0.9879	0.9837	0.9867	0.5912	0.6318
263	H1-hESC	Egr-1	0.2017	0.9666	0.2024	0.9648	0.9536	0.9597	0.1623	0.1849
264	H1-hESC	FOSL1	0.0456	0.9435	0.0409	0.9443	0.9371	0.9395	0.0258	0.0254

265	H1-hESC	GABP	0.4272	0.9871	0.4114	0.9859	0.9828	0.9864	0.3443	0.3881
266	H1-hESC	HDAC2	0.1255	0.9644	0.1103	0.9621	0.9488	0.9586	0.0668	0.1008
267	H1-hESC	JunD	0.2330	0.9703	0.2237	0.9682	0.9571	0.9643	0.1668	0.2119
268	H1-hESC	NANOG	0.1452	0.9545	0.1445	0.9560	0.9304	0.9416	0.0739	0.1101
269	H1-hESC	NRSF	0.5285	0.9627	0.5244	0.9637	0.9529	0.9225	0.4428	0.3701
270	H1-hESC	p300	0.2095	0.9598	0.2043	0.9585	0.9408	0.9532	0.1361	0.1765
271	H1-hESC	Pol2-4H8	0.3586	0.9262	0.3281	0.9196	0.9070	0.9140	0.3090	0.3239
272	H1-hESC	Pol2	0.5428	0.9517	0.5155	0.9468	0.9363	0.9430	0.4972	0.5106
273	H1-hESC	POU5F1	0.2253	0.9649	0.2448	0.9654	0.9471	0.9528	0.1012	0.1497
274	H1-hESC	Rad21	0.7161	0.9818	0.7184	0.9822	0.9740	0.9795	0.6625	0.7030
275	H1-hESC	RXRA	0.0490	0.9785	0.0612	0.9729	0.9561	0.9684	0.0195	0.0293
276	H1-hESC	Sin3Ak-20	0.2747	0.9745	0.2605	0.9712	0.9570	0.9638	0.2033	0.2286
277	H1-hESC	SIX5	0.5097	0.9830	0.5253	0.9744	0.9559	0.9723	0.2870	0.4691
278	H1-hESC	SP1	0.3331	0.9537	0.3196	0.9526	0.9369	0.9468	0.2331	0.2762
279	H1-hESC	SP2	0.3017	0.9748	0.2846	0.9768	0.9717	0.9684	0.2276	0.2826
280	H1-hESC	SP4	0.3144	0.9786	0.3088	0.9787	0.9747	0.9758	0.2884	0.3120
281	H1-hESC	SRF	0.1058	0.8929	0.1171	0.8867	0.8749	0.8897	0.0793	0.1132

282	H1-hESC	TAF1	0.5789	0.9812	0.5545	0.9795	0.9752	0.9786	0.5247	0.5449
283	H1-hESC	TAF7	0.3788	0.9687	0.3493	0.9677	0.9587	0.9652	0.3169	0.3495
284	H1-hESC	TCF12	0.1962	0.9632	0.2173	0.9613	0.9427	0.9543	0.1139	0.1693
285	H1-hESC	TEAD4	0.3389	0.9652	0.3280	0.9619	0.9462	0.9547	0.2208	0.2674
286	H1-hESC	USF1	0.5259	0.9829	0.5308	0.9842	0.9769	0.9787	0.4426	0.4875
287	H1-hESC	YY1	0.4075	0.9711	0.3910	0.9697	0.9558	0.9631	0.3198	0.3605
288	HCT-116	Pol2-4H8	0.4816	0.9253	0.4476	0.9183	0.9040	0.9135	0.4208	0.4479
289	HCT-116	YY1	0.3914	0.9767	0.3633	0.9727	0.9665	0.9688	0.3089	0.3528
290	HCT-116	ZBTB33	0.2495	0.9751	0.2237	0.9701	0.9615	0.9673	0.1717	0.2046
291	HeLa-S3	GABP	0.5112	0.9853	0.4754	0.9852	0.9820	0.9842	0.4157	0.4641
292	HeLa-S3	NRSF	0.4822	0.9615	0.4726	0.9640	0.9410	0.9170	0.3571	0.3467
293	HeLa-S3	Pol2	0.5295	0.9307	0.4939	0.9194	0.9110	0.9208	0.4783	0.4961
294	HeLa-S3	TAF1	0.4143	0.9622	0.3858	0.9580	0.9527	0.9572	0.3628	0.3830
295	HepG2	ATF3	0.3237	0.9898	0.3422	0.9888	0.9842	0.9853	0.2333	0.2951
296	HepG2	BHLHE40	0.1476	0.9816	0.1551	0.9799	0.9701	0.9727	0.1011	0.1402
297	HepG2	CEBPB	0.3756	0.9835	0.3583	0.9839	0.9763	0.9799	0.2961	0.3299
298	HepG2	CEBPD	0.2858	0.9786	0.2562	0.9767	0.9714	0.9735	0.1996	0.2273
299	HepG2	CTCF	0.6849	0.9904	0.6752	0.9905	0.9868	0.9895	0.6385	0.6683
300	HepG2	ELF1	0.4343	0.9761	0.4202	0.9747	0.9657	0.9685	0.3580	0.3883
301	HepG2	FOSL2	0.3673	0.9631	0.3629	0.9605	0.9545	0.9577	0.3106	0.3282
302	HepG2	FOXA1	0.4282	0.9588	0.4276	0.9585	0.9442	0.9480	0.3411	0.3656

303	HepG2	FOXA1	0.4675	0.9582	0.4607	0.9585	0.9435	0.9474	0.3730	0.3986
304	HepG2	FOXA2	0.4242	0.9598	0.4135	0.9590	0.9431	0.9497	0.3282	0.3550
305	HepG2	GABP	0.6518	0.9904	0.6281	0.9901	0.9883	0.9889	0.5616	0.6147
306	HepG2	HDAC2	0.2598	0.9548	0.2360	0.9527	0.9373	0.9428	0.1722	0.1940
307	HepG2	HNF4A	0.3555	0.9738	0.3703	0.9746	0.9620	0.9678	0.2631	0.3226
308	HepG2	HNF4G	0.3522	0.9686	0.3438	0.9685	0.9546	0.9641	0.2527	0.3135
309	HepG2	JunD	0.3284	0.9655	0.3069	0.9622	0.9552	0.9580	0.2614	0.2747
310	HepG2	MBD4	0.1512	0.9331	0.1478	0.9339	0.9169	0.9243	0.0802	0.1018
311	HepG2	MYBL2	0.2848	0.9331	0.2632	0.9318	0.9081	0.9167	0.1959	0.2222
312	HepG2	NFIC	0.2659	0.9359	0.2481	0.9368	0.9087	0.9199	0.1670	0.2000
313	HepG2	NRSF	0.5655	0.9883	0.5489	0.9881	0.9849	0.9730	0.4743	0.4594
314	HepG2	NRSF	0.4773	0.9679	0.4464	0.9653	0.9530	0.9521	0.3508	0.3997
315	HepG2	p300	0.3148	0.9498	0.3037	0.9487	0.9330	0.9334	0.2306	0.2442
316	HepG2	Pol2-4H8	0.4615	0.9268	0.4419	0.9204	0.9084	0.9171	0.3929	0.4276
317	HepG2	Pol2	0.5691	0.9587	0.5344	0.9536	0.9489	0.9521	0.5068	0.5305
318	HepG2	Rad21	0.6674	0.9840	0.6546	0.9840	0.9768	0.9817	0.6042	0.6430
319	HepG2	RXRA	0.2537	0.9559	0.2448	0.9563	0.9420	0.9439	0.1883	0.1932
320	HepG2	Sin3Ak-20	0.3690	0.9640	0.3493	0.9584	0.9507	0.9530	0.3145	0.3324
321	HepG2	SP1	0.3640	0.9584	0.3543	0.9562	0.9427	0.9458	0.2723	0.2981
322	HepG2	SP2	0.4346	0.9942	0.3987	0.9943	0.9917	0.9765	0.3101	0.3469
323	HepG2	SRF	0.0559	0.8965	0.0658	0.8837	0.8537	0.8831	0.0388	0.0488
324	HepG2	TAF1	0.6267	0.9889	0.6013	0.9883	0.9860	0.9878	0.5629	0.5859
325	HepG2	TCF12	0.1186	0.9819	0.1143	0.9811	0.9778	0.9768	0.0673	0.0756
326	HepG2	TEAD4	0.2294	0.9399	0.1972	0.9363	0.9112	0.9182	0.1319	0.1611
327	HepG2	USF1	0.4711	0.9860	0.4627	0.9861	0.9829	0.9838	0.3881	0.4287
328	HepG2	YY1	0.5215	0.9775	0.4946	0.9754	0.9676	0.9729	0.4483	0.4723
329	HepG2	ZBTB33	0.1643	0.9708	0.2309	0.9688	0.9540	0.9651	0.0791	0.1175
330	HepG2	ZBTB7A	0.1907	0.9748	0.1773	0.9741	0.9677	0.9739	0.1377	0.1808
331	HUVEC	Pol2-4H8	0.4521	0.9398	0.4156	0.9333	0.9187	0.9247	0.3772	0.3947
332	HUVEC	Pol2	0.4734	0.9411	0.4410	0.9366	0.9261	0.9318	0.4085	0.4307
333	K562	ATF3	0.3178	0.9771	0.2999	0.9757	0.9665	0.9722	0.2484	0.2857
334	K562	BCL3	0.1388	0.9590	0.1224	0.9554	0.9468	0.9554	0.0554	0.1089
335	K562	BCLAF1	0.1747	0.9589	0.1584	0.9584	0.9520	0.9591	0.1166	0.1640
336	K562	CBX3	0.2060	0.9037	0.1915	0.9011	0.8675	0.8849	0.1353	0.1621

337	K562	CEBPB	0.3480	0.9637	0.3116	0.9616	0.9453	0.9551	0.2333	0.2804
338	K562	CTCF	0.6488	0.9877	0.6371	0.9872	0.9838	0.9859	0.6037	0.6289
339	K562	CTCFL	0.3543	0.9917	0.3319	0.9914	0.9899	0.9911	0.2861	0.3366
340	K562	E2F6	0.4142	0.9686	0.4012	0.9667	0.9632	0.9672	0.3782	0.4042
341	K562	Egr-1	0.3593	0.9579	0.3502	0.9586	0.9315	0.9367	0.2728	0.2931
342	K562	ELF1	0.4702	0.9703	0.4547	0.9693	0.9583	0.9641	0.3942	0.4251
343	K562	ETS1	0.4128	0.9823	0.4006	0.9802	0.9744	0.9797	0.3350	0.3924
344	K562	FOSL1	0.3319	0.9824	0.3114	0.9817	0.9779	0.9800	0.3003	0.2978
345	K562	GABP	0.5211	0.9788	0.4999	0.9776	0.9706	0.9756	0.4303	0.4910
346	K562	GATA2	0.3244	0.9669	0.3011	0.9659	0.9583	0.9609	0.2557	0.2721
347	K562	HDAC2	0.1633	0.9552	0.1439	0.9495	0.9469	0.9535	0.1008	0.1209
348	K562	Max	0.5115	0.9590	0.4889	0.9564	0.9465	0.9541	0.4420	0.4795
349	K562	MEF2A	0.1076	0.9485	0.0930	0.9491	0.9166	0.9186	0.0486	0.0568
350	K562	NR2F2	0.2238	0.9372	0.2108	0.9354	0.9210	0.9220	0.1707	0.1789
351	K562	NRSF	0.4014	0.9639	0.3871	0.9615	0.9506	0.9362	0.2875	0.2976
352	K562	PML	0.3870	0.9535	0.3551	0.9492	0.9427	0.9483	0.3120	0.3412
353	K562	Pol2-4H8	0.4189	0.9449	0.3787	0.9420	0.9318	0.9395	0.3381	0.3724
354	K562	Pol2	0.6259	0.9611	0.5886	0.9572	0.9513	0.9573	0.5694	0.5941
355	K562	PU.1	0.3794	0.9780	0.3689	0.9786	0.9679	0.9695	0.2723	0.2956
356	K562	Rad21	0.6167	0.9912	0.5978	0.9906	0.9875	0.9898	0.5605	0.5880
357	K562	Sin3Ak-20	0.3694	0.9809	0.3267	0.9762	0.9738	0.9753	0.2868	0.3148
358	K562	SIX5	0.5485	0.9942	0.5476	0.9934	0.9917	0.9926	0.4014	0.5278
359	K562	SP1	0.3332	0.9810	0.3166	0.9764	0.9727	0.9766	0.2705	0.3052
360	K562	SP2	0.4186	0.9894	0.4061	0.9864	0.9912	0.9903	0.3254	0.3980
361	K562	SRF	0.1277	0.9288	0.1270	0.9182	0.9140	0.9274	0.0896	0.1140
362	K562	STAT5A	0.2582	0.9610	0.2371	0.9593	0.9482	0.9554	0.1697	0.2054
363	K562	TAF1	0.5074	0.9792	0.4732	0.9776	0.9734	0.9760	0.4397	0.4774
364	K562	TAF7	0.1375	0.9084	0.1099	0.9091	0.8904	0.9002	0.0646	0.0877
365	K562	TEAD4	0.3243	0.9402	0.3021	0.9342	0.9226	0.9281	0.2557	0.2725
366	K562	THAP1	0.2256	0.9880	0.2230	0.9878	0.9858	0.9871	0.1912	0.2039
367	K562	TRIM28	0.2193	0.9356	0.1903	0.9325	0.9168	0.9255	0.1366	0.1702
368	K562	USF1	0.4475	0.9823	0.4335	0.9824	0.9746	0.9784	0.3730	0.4044
369	K562	YY1	0.5119	0.9880	0.4837	0.9868	0.9830	0.9855	0.4250	0.4591
370	K562	YY1	0.4361	0.9657	0.4240	0.9663	0.9574	0.9615	0.3689	0.3818

371	K562	ZBTB33	0.1727	0.9673	0.2651	0.9704	0.9558	0.9655	0.0635	0.1180
372	K562	ZBTB7A	0.4341	0.9749	0.4148	0.9733	0.9704	0.9730	0.3732	0.4183
373	PANC-1	NRSF	0.5320	0.9844	0.5159	0.9829	0.9751	0.9662	0.4056	0.4254
374	PANC-1	Pol2-4H8	0.2168	0.9397	0.2046	0.9378	0.9290	0.9367	0.1696	0.1863
375	PANC-1	Sin3Ak-20	0.2054	0.9867	0.1878	0.9836	0.9803	0.9829	0.1697	0.1798
376	PFSK-1	FOXP2	0.3701	0.9691	0.3541	0.9674	0.9624	0.9661	0.3299	0.3499
377	PFSK-1	NRSF	0.4709	0.9574	0.4543	0.9545	0.9351	0.9085	0.3528	0.3326
378	PFSK-1	Sin3Ak-20	0.1346	0.9519	0.1055	0.9447	0.9302	0.9386	0.0799	0.0937
379	PFSK-1	TAF1	0.3026	0.9391	0.2764	0.9346	0.9223	0.9325	0.2167	0.2512
380	SK-N-MC	FOXP2	0.2846	0.9259	0.2717	0.9251	0.9102	0.9230	0.2325	0.2663
381	SK-N-MC	Pol2-4H8	0.3549	0.9302	0.3256	0.9249	0.9071	0.9227	0.2610	0.3172
382	SK-N-SH	NRSF	0.4046	0.9719	0.3819	0.9681	0.9580	0.9620	0.2923	0.3503
383	SK-N-SH	NRSF	0.4055	0.9763	0.3794	0.9724	0.9591	0.9491	0.2845	0.3130
384	SK-N-SH	Pol2-4H8	0.3693	0.9273	0.3404	0.9188	0.9016	0.9112	0.3011	0.3228
385	SK-N-SH_RA	CTCF	0.6809	0.9936	0.6548	0.9933	0.9910	0.9925	0.6051	0.6568
386	SK-N-SH_RA	p300	0.2981	0.9177	0.2725	0.9108	0.8919	0.9021	0.2222	0.2483
387	SK-N-SH_RA	Rad21	0.6102	0.9694	0.5913	0.9677	0.9581	0.9654	0.5440	0.5832
388	SK-N-SH_RA	USF1	0.4684	0.9843	0.4628	0.9843	0.9781	0.9803	0.3890	0.4282
389	SK-N-SH_RA	YY1	0.3869	0.9598	0.3706	0.9568	0.9447	0.9527	0.3019	0.3336
390	SK-N-SH	Sin3Ak-20	0.3397	0.9607	0.3162	0.9541	0.9469	0.9499	0.2882	0.2975
391	SK-N-SH	TAF1	0.4831	0.9771	0.4524	0.9738	0.9690	0.9711	0.4097	0.4396
392	T-47D	CTCF	0.7422	0.9919	0.7155	0.9910	0.9872	0.9897	0.6689	0.7066

393	T-47D	ERalpha	0.0387	0.9098	0.0361	0.9133	0.8954	0.8929	0.0302	0.0329
394	T-47D	ERalpha	0.0966	0.8949	0.0817	0.8966	0.8772	0.8762	0.0642	0.0757
395	T-47D	ERalpha	0.0955	0.8938	0.0797	0.8953	0.8772	0.8769	0.0638	0.0758
396	T-47D	FOXA1	0.3282	0.9395	0.3192	0.9396	0.9181	0.9285	0.2447	0.2866
397	T-47D	GATA3	0.2427	0.9052	0.2400	0.9056	0.8646	0.8705	0.1495	0.1673
398	T-47D	p300	0.1653	0.9203	0.1436	0.9152	0.8826	0.9047	0.1005	0.1281
399	U87	NRSF	0.4987	0.9828	0.4801	0.9832	0.9778	0.9752	0.3989	0.4430
400	U87	Pol2-4H8	0.4076	0.9341	0.3777	0.9283	0.9165	0.9245	0.3459	0.3712
401	A549	BHLHE40	0.1771	0.9694	0.1744	0.9631	0.9584	0.9629	0.1239	0.1621
402	A549	CEBPB	0.5708	0.9795	0.5588	0.9785	0.9716	0.9745	0.4908	0.5156
403	A549	Max	0.4045	0.9788	0.3850	0.9776	0.9701	0.9754	0.3150	0.3530
404	A549	ol2(phosphoS2	0.0228	0.7566	0.0118	0.7116	0.6639	0.6974	0.0085	0.0107
405	A549	Rad21	0.7165	0.9918	0.6844	0.9910	0.9886	0.9904	0.6366	0.6790
406	GM08714	ZNF274	0.5153	0.9844	0.4869	0.9794	0.9720	0.9721	0.5179	0.5778
407	GM10847	NFKB	0.2767	0.9662	0.2329	0.9611	0.9515	0.9529	0.1596	0.2030
408	GM10847	Pol2	0.6087	0.9927	0.5825	0.9915	0.9917	0.9917	0.5294	0.5760
409	GM12878	BHLHE40	0.2815	0.9577	0.2496	0.9537	0.9443	0.9490	0.2205	0.2329
410	GM12878	BRCA1	0.3671	0.9976	0.5929	0.9995	0.9973	0.9977	0.0575	0.1568
411	GM12878	c-Fos	0.5074	0.9961	0.4407	0.9910	0.9888	0.9921	0.4087	0.4286
412	GM12878	CHD1	0.0919	0.9050	0.0795	0.8908	0.8884	0.8926	0.0706	0.0776
413	GM12878	CHD2	0.3785	0.9549	0.3420	0.9503	0.9420	0.9445	0.2990	0.3266
414	GM12878	COREST	0.1385	0.9798	0.1544	0.9738	0.9608	0.9698	0.0523	0.0799
415	GM12878	CTCF	0.6255	0.9854	0.6238	0.9853	0.9790	0.9840	0.5500	0.6069

416	GM12878	E2F4	0.2327	0.9715	0.2031	0.9736	0.9707	0.9762	0.1961	0.2310
417	GM12878	EBF1	0.3182	0.9298	0.3009	0.9251	0.8925	0.9266	0.1873	0.2908
418	GM12878	ELK1	0.4795	0.9699	0.4573	0.9663	0.9657	0.9674	0.4044	0.4367
419	GM12878	IKZF1	0.1824	0.9354	0.1697	0.9322	0.9237	0.9296	0.1335	0.1472
420	GM12878	JunD	0.2093	0.9772	0.1551	0.9789	0.9662	0.9720	0.1017	0.1199
421	GM12878	Max	0.4134	0.9636	0.3761	0.9592	0.9575	0.9590	0.3424	0.3752
422	GM12878	MAZ	0.4637	0.9614	0.4380	0.9567	0.9520	0.9551	0.4059	0.4360
423	GM12878	Mxi1	0.4073	0.9494	0.3748	0.9436	0.9393	0.9434	0.3382	0.3681
424	GM12878	NF-E2	0.4472	0.9938	0.4565	0.9879	0.9857	0.9892	0.3453	0.4321
425	GM12878	NFKB	0.3112	0.9540	0.2732	0.9488	0.9398	0.9411	0.2170	0.2397
426	GM12878	NF-YA	0.4728	0.9899	0.4736	0.9915	0.9872	0.9867	0.4052	0.4643
427	GM12878	NF-YB	0.4757	0.9564	0.4607	0.9580	0.9532	0.9591	0.4111	0.4579
428	GM12878	Nrf1	0.5559	0.9911	0.5529	0.9916	0.9880	0.9916	0.4514	0.5275
429	GM12878	p300	0.2452	0.9683	0.2009	0.9676	0.9600	0.9605	0.1496	0.1664
430	GM12878	p300	0.2829	0.9444	0.2475	0.9399	0.9319	0.9306	0.2057	0.2142
431	GM12878	Pol2	0.4385	0.9294	0.4114	0.9227	0.9195	0.9213	0.3835	0.4023
432	GM12878	Pol2(phosphoS2	0.3823	0.9330	0.3655	0.9255	0.9241	0.9272	0.3403	0.3715

433	GM12878	Pol2	0.4638	0.9734	0.4321	0.9682	0.9681	0.9694	0.4035	0.4310
434	GM12878	Pol3	0.0049	0.9758	0.0056	0.8952	0.9421	0.9867	0.0001	0.0037
435	GM12878	Rad21	0.6628	0.9852	0.6463	0.9845	0.9792	0.9832	0.5944	0.6355
436	GM12878	RFX5	0.2409	0.9343	0.2333	0.9343	0.9212	0.9337	0.2089	0.2309
437	GM12878	SIN3A	0.3905	0.9582	0.3585	0.9532	0.9500	0.9545	0.3272	0.3537
438	GM12878	SMC3	0.7238	0.9825	0.7016	0.9804	0.9771	0.9792	0.6670	0.6983
439	GM12878	STAT1	0.0756	0.9322	0.0487	0.9376	0.9255	0.9260	0.0623	0.0574
440	GM12878	STAT3	0.1320	0.9253	0.1002	0.9182	0.9116	0.9191	0.0865	0.0961
441	GM12878	TBLR1	0.2581	0.9423	0.2202	0.9344	0.9273	0.9313	0.1834	0.2023
442	GM12878	TBP	0.2820	0.9228	0.2476	0.9161	0.9114	0.9132	0.2201	0.2360
443	GM12878	TR4	0.1745	0.9665	0.1471	0.9701	0.9623	0.9663	0.1450	0.1442
444	GM12878	USF2	0.4191	0.9799	0.3995	0.9789	0.9731	0.9764	0.3246	0.3621
445	GM12878	WHIP	0.1368	0.8955	0.1148	0.8794	0.8718	0.8803	0.1005	0.1191
446	GM12878	YY1	0.1523	0.9491	0.1159	0.9397	0.9267	0.9332	0.0744	0.0891
447	GM12878	Znf143	0.6959	0.9903	0.6670	0.9897	0.9826	0.9878	0.6168	0.6689
448	GM12878	ZNF274	0.0296	0.8205	0.0320	0.8027	0.7069	0.7413	0.0019	0.0292
449	GM12878	ZZZ3	0.2819	0.9640	0.3213	0.9383	0.9083	0.9319	0.0340	0.1253

450	GM12891	NFKB	0.4216	0.9537	0.3912	0.9506	0.9382	0.9423	0.2978	0.3294
451	GM12891	Pol2	0.4396	0.9330	0.4114	0.9265	0.9242	0.9283	0.3716	0.4011
452	GM12892	NFKB	0.2907	0.9706	0.2523	0.9650	0.9562	0.9577	0.1681	0.2131
453	GM12892	Pol2	0.5414	0.9546	0.5115	0.9506	0.9481	0.9499	0.4807	0.5040
454	GM15510	NFKB	0.3443	0.9620	0.3157	0.9571	0.9483	0.9527	0.2632	0.2896
455	GM15510	Pol2	0.6484	0.9765	0.6204	0.9752	0.9744	0.9746	0.5932	0.6160
456	GM18505	NFKB	0.2966	0.9595	0.2391	0.9541	0.9436	0.9441	0.1563	0.1956
457	GM18505	Pol2	0.4906	0.9434	0.4582	0.9379	0.9363	0.9400	0.4406	0.4610
458	GM18526	NFKB	0.1932	0.9777	0.1603	0.9706	0.9585	0.9590	0.0749	0.1082
459	GM18526	Pol2	0.6112	0.9784	0.5825	0.9760	0.9753	0.9756	0.5479	0.5753
460	GM18951	NFKB	0.3064	0.9571	0.2648	0.9529	0.9419	0.9433	0.1983	0.2277
461	GM18951	Pol2	0.6479	0.9706	0.6215	0.9684	0.9660	0.9677	0.5960	0.6145
462	GM19099	NFKB	0.2883	0.9726	0.2538	0.9681	0.9598	0.9581	0.1636	0.2022
463	GM19099	Pol2	0.5333	0.9560	0.5029	0.9526	0.9484	0.9512	0.4737	0.5014
464	GM19193	NFKB	0.2885	0.9791	0.2352	0.9758	0.9671	0.9646	0.1412	0.1892
465	GM19193	Pol2	0.5740	0.9624	0.5451	0.9603	0.9579	0.9597	0.5155	0.5360
466	H1-hESC	Bach1	0.2952	0.9694	0.2916	0.9680	0.9524	0.9557	0.2039	0.2371

467	H1-hESC	BRCA1	0.1177	0.9698	0.2001	0.9716	0.9508	0.9523	0.0862	0.1132
468	H1-hESC	CEBPB	0.4826	0.9847	0.4949	0.9851	0.9734	0.9805	0.3280	0.4361
469	H1-hESC	CHD1	0.0597	0.9542	0.0529	0.9540	0.9465	0.9519	0.0598	0.0640
470	H1-hESC	CHD2	0.3178	0.9787	0.3162	0.9771	0.9676	0.9730	0.2552	0.2796
471	H1-hESC	c-Jun	0.2389	0.9835	0.2544	0.9802	0.9607	0.9718	0.0995	0.1971
472	H1-hESC	c-Myc	0.3009	0.9869	0.2798	0.9851	0.9718	0.9813	0.1767	0.2109
473	H1-hESC	CtBP2	0.1926	0.9602	0.1826	0.9616	0.9508	0.9586	0.1523	0.1721
474	H1-hESC	GTF2F1	0.2596	0.9744	0.2377	0.9766	0.9585	0.9660	0.2061	0.2339
475	H1-hESC	JunD	0.3048	0.9716	0.3247	0.9695	0.9495	0.9634	0.2191	0.2781
476	H1-hESC	MafK	0.4769	0.9802	0.4535	0.9786	0.9702	0.9686	0.3372	0.3589
477	H1-hESC	Max	0.3487	0.9669	0.3482	0.9687	0.9468	0.9636	0.2344	0.2944
478	H1-hESC	Mxi1	0.3409	0.9803	0.3351	0.9792	0.9689	0.9754	0.2186	0.2737
479	H1-hESC	Nrf1	0.5384	0.9949	0.5454	0.9950	0.9929	0.9932	0.3950	0.4903
480	H1-hESC	Rad21	0.6691	0.9856	0.6694	0.9856	0.9800	0.9839	0.6186	0.6559
481	H1-hESC	RFX5	0.0339	0.8864	0.0285	0.8877	0.8495	0.8804	0.0285	0.0279
482	H1-hESC	SIN3A	0.4342	0.9639	0.4169	0.9620	0.9465	0.9577	0.3635	0.3974
483	H1-hESC	SUZ12	0.2956	0.9826	0.2830	0.9814	0.9776	0.9809	0.2516	0.2825

484	H1-hESC	TBP	0.4484	0.9668	0.4278	0.9660	0.9580	0.9635	0.3928	0.4222
485	H1-hESC	USF2	0.4804	0.9872	0.4736	0.9888	0.9832	0.9860	0.3673	0.4280
486	H1-hESC	Znf143	0.6276	0.9687	0.6266	0.9670	0.9530	0.9628	0.5380	0.5999
487	HCT-116	Pol2	0.5450	0.9627	0.5178	0.9582	0.9545	0.9554	0.4872	0.5096
488	HCT-116	TCF7L2	0.2661	0.9148	0.2590	0.9206	0.8983	0.9110	0.2237	0.2503
489	HEK293	ELK4	0.1622	0.9226	0.1719	0.8996	0.8969	0.8950	0.1974	0.1544
490	HEK293	KAP1	0.1711	0.8618	0.1546	0.8486	0.8172	0.8388	0.1123	0.1448
491	HEK293	Pol2	0.6113	0.9946	0.5858	0.9932	0.9914	0.9935	0.5293	0.5733
492	HEK293	TCF7L2	0.1021	0.8959	0.0969	0.8882	0.8630	0.8764	0.0695	0.0819
493	HEK293-T-REx	ZNF263	0.4036	0.9398	0.4012	0.9412	0.8740	0.8795	0.2363	0.2456
494	HeLa-S3	AP-2alpha	0.3089	0.9446	0.2779	0.9365	0.9012	0.9269	0.1954	0.2534
495	HeLa-S3	AP-2gamma	0.3345	0.9370	0.3035	0.9300	0.8973	0.9214	0.2297	0.2796
496	HeLa-S3	BAF155	0.2054	0.9351	0.1837	0.9293	0.9095	0.9208	0.1469	0.1725
497	HeLa-S3	BAF170	0.1094	0.9583	0.1094	0.9470	0.9285	0.9452	0.0701	0.0977
498	HeLa-S3	BDP1	0.1458	0.9156	0.1127	0.9234	0.9123	0.8797	0.0095	0.1092
499	HeLa-S3	BRCA1	0.2769	0.9634	0.2696	0.9585	0.9448	0.9537	0.2265	0.2457
500	HeLa-S3	BRF1	0.0001	0.7278	0.0001	0.6609	0.8307	0.6870	0.0001	0.0002
501	HeLa-S3	BRF2	0.0122	0.8487	0.1185	0.8291	0.7953	0.8319	0.0053	0.1183
502	HeLa-S3	Brg1	0.0307	0.9193	0.0335	0.9114	0.8915	0.9070	0.0224	0.0309

503	HeLa-S3	CEBPB	0.4553	0.9513	0.4385	0.9487	0.9356	0.9405	0.3850	0.3999
504	HeLa-S3	c-Fos	0.3276	0.9824	0.2988	0.9795	0.9740	0.9779	0.2478	0.2578
505	HeLa-S3	CHD2	0.4342	0.9593	0.4074	0.9544	0.9452	0.9508	0.3775	0.3995
506	HeLa-S3	c-Jun	0.4047	0.9649	0.3795	0.9632	0.9498	0.9562	0.3220	0.3388
507	HeLa-S3	c-Myc	0.3066	0.9700	0.2785	0.9653	0.9545	0.9608	0.2360	0.2478
508	HeLa-S3	COREST	0.2900	0.9400	0.2636	0.9343	0.9151	0.9288	0.2240	0.2486
509	HeLa-S3	E2F1	0.3036	0.9868	0.2915	0.9862	0.9867	0.9876	0.2729	0.3112
510	HeLa-S3	E2F4	0.2218	0.9741	0.2107	0.9663	0.9698	0.9703	0.1977	0.2349
511	HeLa-S3	E2F6	0.2936	0.9773	0.2851	0.9764	0.9711	0.9749	0.2399	0.2669
512	HeLa-S3	ELK1	0.4877	0.9768	0.4652	0.9730	0.9743	0.9749	0.4202	0.4584
513	HeLa-S3	ELK4	0.4411	0.9745	0.4304	0.9730	0.9621	0.9674	0.3619	0.3973
514	HeLa-S3	GTF2F1	0.3143	0.9481	0.2760	0.9418	0.9312	0.9375	0.2413	0.2690
515	HeLa-S3	HA-E2F1	0.4409	0.9917	0.4254	0.9901	0.9910	0.9916	0.4121	0.4408
516	HeLa-S3	Ini1	0.2029	0.9668	0.1975	0.9640	0.9581	0.9625	0.1785	0.1876
517	HeLa-S3	IRF3	0.5333	0.9914	0.4798	0.9889	0.9870	0.9838	0.4166	0.4673
518	HeLa-S3	JunD	0.4335	0.9635	0.4082	0.9622	0.9498	0.9560	0.3628	0.3802
519	HeLa-S3	MafK	0.3785	0.9670	0.3510	0.9652	0.9513	0.9527	0.2592	0.2799

520	HeLa-S3	Max	0.4263	0.9492	0.3915	0.9446	0.9297	0.9370	0.3546	0.3821
521	HeLa-S3	MAZ	0.4488	0.9728	0.4409	0.9710	0.9665	0.9695	0.3944	0.4316
522	HeLa-S3	Mxi1	0.3658	0.9629	0.3311	0.9551	0.9481	0.9533	0.3092	0.3232
523	HeLa-S3	NF-YA	0.4281	0.9744	0.3983	0.9733	0.9685	0.9767	0.3366	0.3961
524	HeLa-S3	NF-YB	0.3541	0.9592	0.3370	0.9591	0.9487	0.9581	0.2714	0.3304
525	HeLa-S3	Nrf1	0.5197	0.9754	0.5307	0.9784	0.9740	0.9818	0.3811	0.4552
526	HeLa-S3	p300	0.3328	0.9458	0.3099	0.9418	0.9233	0.9303	0.2616	0.2723
527	HeLa-S3	Pol2(phosphoS2	0.3690	0.9226	0.3424	0.9130	0.9099	0.9155	0.3250	0.3395
528	HeLa-S3	Pol2	0.6035	0.9641	0.5719	0.9588	0.9561	0.9590	0.5722	0.5851
529	HeLa-S3	PRDM1	0.0694	0.9275	0.0588	0.9237	0.8976	0.9084	0.0425	0.0458
530	HeLa-S3	Rad21	0.5896	0.9734	0.5672	0.9729	0.9613	0.9687	0.5203	0.5594
531	HeLa-S3	RFX5	0.3217	0.9408	0.2930	0.9370	0.9220	0.9321	0.2559	0.2853
532	HeLa-S3	RPC155	0.1371	0.9362	0.1471	0.9299	0.9191	0.9313	0.0588	0.0951
533	HeLa-S3	SMC3	0.5504	0.9715	0.5294	0.9706	0.9617	0.9670	0.4864	0.5227
534	HeLa-S3	SPT20	0.0063	0.8191	0.0074	0.8258	0.7839	0.8193	0.0046	0.0064
535	HeLa-S3	STAT1	0.2704	0.9208	0.2441	0.9151	0.8775	0.9093	0.1622	0.2229
536	HeLa-S3	STAT3	0.3043	0.9555	0.2729	0.9516	0.9336	0.9414	0.2136	0.2295

537	HeLa-S3	TBP	0.3211	0.9318	0.2866	0.9229	0.9122	0.9182	0.2626	0.2774
538	HeLa-S3	TCF7L2	0.2401	0.8969	0.2294	0.8949	0.8671	0.8833	0.1833	0.2046
539	HeLa-S3	TCF7L2	0.0642	0.9077	0.0638	0.9086	0.8696	0.8871	0.0451	0.0584
540	HeLa-S3	TFIIIC-110	0.1136	0.9494	0.1192	0.9517	0.9353	0.9477	0.0729	0.1081
541	HeLa-S3	TR4	0.0951	0.9178	0.0721	0.9067	0.8902	0.9093	0.0522	0.0689
542	HeLa-S3	USF2	0.3376	0.9661	0.3304	0.9636	0.9544	0.9582	0.2730	0.3011
543	HeLa-S3	ZKSCAN1	0.1139	0.9120	0.1105	0.9167	0.9070	0.9168	0.0992	0.1050
544	HeLa-S3	Znf143	0.3973	0.9760	0.3875	0.9737	0.9601	0.9701	0.3091	0.3768
545	HeLa-S3	ZNF274	0.0001	0.9604	0.0001	0.9061	0.9040	0.9488	0.0002	0.0001
546	HeLa-S3	ZZZ3	0.0815	0.9333	0.0404	0.9236	0.8552	0.8719	0.0102	0.0192
547	HepG2	ARID3A	0.2452	0.9482	0.2222	0.9435	0.9247	0.9326	0.1553	0.1954
548	HepG2	BHLHE40	0.2763	0.9655	0.2690	0.9622	0.9509	0.9575	0.2236	0.2507
549	HepG2	BRCA1	0.2068	0.9911	0.3645	0.9926	0.9862	0.9834	0.1195	0.1765
550	HepG2	CEBPB	0.4124	0.9823	0.3974	0.9813	0.9724	0.9762	0.3136	0.3492
551	HepG2	CEBPB	0.6164	0.9831	0.6154	0.9834	0.9771	0.9786	0.5495	0.5783
552	HepG2	CHD2	0.2253	0.9775	0.2629	0.9793	0.9703	0.9744	0.1696	0.1735
553	HepG2	c-Jun	0.4195	0.9836	0.3965	0.9837	0.9715	0.9787	0.2930	0.3534
554	HepG2	COREST	0.1802	0.9672	0.1919	0.9660	0.9545	0.9628	0.1247	0.1429
555	HepG2	ERRA	0.0577	0.9788	0.0744	0.9807	0.9744	0.9722	0.0444	0.0442
556	HepG2	GRp20	0.2725	0.9974	0.2446	0.9947	0.9957	0.9883	0.1771	0.1694
557	HepG2	HNF4A	0.3334	0.9762	0.3236	0.9782	0.9653	0.9751	0.2165	0.2737
558	HepG2	HSF1	0.1722	0.9617	0.1254	0.9552	0.9384	0.9547	0.0277	0.1180
559	HepG2	IRF3	0.4609	0.9838	0.4309	0.9778	0.9625	0.9796	0.3506	0.3834
560	HepG2	JunD	0.6186	0.9836	0.6215	0.9842	0.9714	0.9790	0.4573	0.5635

561	HepG2	MafF	0.6024	0.9843	0.6021	0.9838	0.9762	0.9741	0.5173	0.5211
562	HepG2	MafK	0.7648	0.9889	0.7685	0.9888	0.9822	0.9770	0.6808	0.6745
563	HepG2	MafK	0.6456	0.9870	0.6426	0.9866	0.9789	0.9736	0.5428	0.5361
564	HepG2	Max	0.4129	0.9814	0.3909	0.9811	0.9754	0.9788	0.3377	0.3671
565	HepG2	MAZ	0.4370	0.9808	0.4226	0.9778	0.9755	0.9782	0.3762	0.4058
566	HepG2	Mxi1	0.4912	0.9735	0.4611	0.9718	0.9625	0.9678	0.4091	0.4385
567	HepG2	Nrf1	0.5839	0.9916	0.5884	0.9900	0.9936	0.9891	0.3688	0.5116
568	HepG2	p300	0.2018	0.9639	0.1817	0.9605	0.9471	0.9562	0.1131	0.1429
569	HepG2	PGC1A	0.0466	0.9447	0.0407	0.9382	0.9239	0.9326	0.0180	0.0405
570	HepG2	Pol2	0.4394	0.9638	0.4322	0.9609	0.9580	0.9616	0.4004	0.4238
571	HepG2	Pol2	0.4596	0.9551	0.4323	0.9497	0.9403	0.9453	0.4019	0.4260
572	HepG2	ol2(phosphoS2	0.1199	0.8836	0.0875	0.8568	0.8449	0.8582	0.0775	0.0810
573	HepG2	Rad21	0.6395	0.9878	0.6267	0.9877	0.9819	0.9855	0.5839	0.6189
574	HepG2	RFX5	0.2611	0.9218	0.2438	0.9173	0.8976	0.9128	0.2369	0.2451
575	HepG2	SMC3	0.6595	0.9869	0.6331	0.9859	0.9798	0.9836	0.5949	0.6251
576	HepG2	SREBP1	0.2571	0.9863	0.2663	0.9852	0.9809	0.9832	0.1769	0.1935
577	HepG2	TBP	0.4038	0.9640	0.3800	0.9606	0.9505	0.9557	0.3415	0.3581
578	HepG2	TCF7L2	0.0829	0.9266	0.0750	0.9318	0.8994	0.9038	0.0452	0.0502
579	HepG2	TR4	0.2168	0.9661	0.1930	0.9643	0.9554	0.9625	0.1619	0.1720
580	HepG2	USF2	0.4254	0.9918	0.4227	0.9923	0.9887	0.9891	0.3234	0.3635
581	HepG2	ZNF274	0.0001	0.7696	0.0000	0.6715	0.5815	0.6671	0.0000	0.0000
582	HUVEC	c-Fos	0.5154	0.9589	0.4981	0.9566	0.9430	0.9488	0.4358	0.4582
583	HUVEC	c-Jun	0.4172	0.9709	0.3858	0.9674	0.9548	0.9597	0.3316	0.3441
584	HUVEC	GATA2	0.3592	0.9315	0.3346	0.9288	0.9038	0.9065	0.2606	0.2780
585	HUVEC	Max	0.3638	0.9875	0.3569	0.9857	0.9822	0.9853	0.2981	0.3289
586	HUVEC	Pol2	0.3440	0.9366	0.3196	0.9292	0.9197	0.9263	0.2875	0.2977
587	IMR90	CEBPB	0.5934	0.9708	0.5765	0.9694	0.9577	0.9638	0.4919	0.5421
588	IMR90	CTCF	0.7065	0.9869	0.6923	0.9867	0.9815	0.9843	0.6523	0.6799
589	IMR90	MafK	0.5765	0.9786	0.5646	0.9776	0.9696	0.9657	0.4775	0.4809
590	IMR90	Pol2	0.4256	0.9458	0.4001	0.9376	0.9278	0.9372	0.3818	0.4024
591	IMR90	Rad21	0.6072	0.9803	0.5771	0.9790	0.9725	0.9767	0.5414	0.5734
592	K562	ARID3A	0.1613	0.9490	0.1379	0.9448	0.9341	0.9391	0.1087	0.1251
593	K562	ATF1	0.3121	0.9747	0.2962	0.9736	0.9634	0.9672	0.2570	0.2712
594	K562	ATF3	0.3657	0.9880	0.3323	0.9846	0.9739	0.9828	0.2727	0.3046

595	K562	Bach1	0.2752	0.9651	0.2738	0.9617	0.9508	0.9436	0.1822	0.1827
596	K562	BDP1	0.3108	0.9796	0.3660	0.9958	0.9629	0.9856	0.0244	0.1445
597	K562	BHLHE40	0.3070	0.9559	0.3007	0.9554	0.9472	0.9536	0.2525	0.2782
599	K562	BRF2	0.0045	0.7924	0.0021	0.7813	0.7482	0.7725	0.0010	0.0015
600	K562	Brg1	0.0786	0.9336	0.0745	0.9267	0.9186	0.9218	0.0480	0.0859
601	K562	CCNT2	0.4927	0.9713	0.4737	0.9707	0.9653	0.9686	0.4608	0.4762
602	K562	CEBPB	0.4684	0.9729	0.4425	0.9716	0.9607	0.9666	0.3613	0.4151
603	K562	c-Fos	0.4241	0.9885	0.4063	0.9874	0.9856	0.9886	0.3027	0.3863
604	K562	CHD2	0.3448	0.9771	0.3755	0.9758	0.9644	0.9725	0.2505	0.2900
605	K562	c-Jun	0.3031	0.9771	0.2796	0.9755	0.9645	0.9714	0.2360	0.2539
606	K562	c-Jun	0.2765	0.9828	0.2510	0.9789	0.9732	0.9764	0.2102	0.2369
607	K562	c-Jun	0.3022	0.9774	0.2738	0.9735	0.9661	0.9688	0.2381	0.2524
608	K562	c-Jun	0.3124	0.9786	0.2785	0.9774	0.9700	0.9731	0.2421	0.2583
609	K562	c-Jun	0.3204	0.9883	0.2975	0.9858	0.9810	0.9844	0.2611	0.2636
610	K562	c-Myc	0.3479	0.9758	0.3267	0.9752	0.9665	0.9728	0.2520	0.2922
611	K562	c-Myc	0.3518	0.9690	0.3361	0.9701	0.9592	0.9651	0.2637	0.3017
612	K562	c-Myc	0.4576	0.9590	0.4229	0.9569	0.9450	0.9523	0.3692	0.4129
613	K562	c-Myc	0.4001	0.9595	0.3670	0.9573	0.9442	0.9525	0.3079	0.3419
614	K562	c-Myc	0.4183	0.9603	0.3891	0.9558	0.9403	0.9515	0.3351	0.3728
615	K562	c-Myc	0.3031	0.9880	0.2702	0.9854	0.9781	0.9848	0.2002	0.2393
616	K562	COREST	0.1406	0.9322	0.1245	0.9331	0.9085	0.9216	0.0908	0.1114
617	K562	COREST	0.2964	0.9295	0.2805	0.9299	0.9106	0.9199	0.2253	0.2588
618	K562	CTCF	0.6293	0.9863	0.6175	0.9855	0.9812	0.9842	0.5696	0.6092
619	K562	E2F4	0.4550	0.9926	0.4290	0.9919	0.9911	0.9917	0.4184	0.4402
620	K562	E2F6	0.3743	0.9730	0.3615	0.9701	0.9651	0.9702	0.3305	0.3482
621	K562	ELK1	0.4881	0.9781	0.4676	0.9779	0.9746	0.9785	0.4137	0.4441
622	K562	GATA1	0.3035	0.9824	0.2597	0.9835	0.9781	0.9807	0.1682	0.2216
623	K562	GATA2	0.2926	0.9709	0.2603	0.9707	0.9612	0.9612	0.2127	0.2435
624	K562	GTF2B	0.1778	0.9590	0.1522	0.9575	0.9543	0.9542	0.1176	0.1201
625	K562	GTF2F1	0.2064	0.9641	0.1859	0.9613	0.9651	0.9678	0.1711	0.1635
626	K562	HMGN3	0.5022	0.9804	0.4709	0.9794	0.9763	0.9782	0.4622	0.4760
627	K562	Ini1	0.0311	0.9217	0.0321	0.9239	0.9019	0.9072	0.0336	0.0385
628	K562	IRF1	0.1809	0.9693	0.1601	0.9717	0.9510	0.9515	0.0386	0.1143
629	K562	IRF1	0.3224	0.9776	0.2832	0.9736	0.9590	0.9702	0.1847	0.2504

630	K562	IRF1	0.3291	0.9646	0.2926	0.9644	0.9488	0.9587	0.2302	0.2748
631	K562	IRF1	0.6012	0.9867	0.5661	0.9855	0.9809	0.9853	0.5501	0.5710
632	K562	JunD	0.4257	0.9609	0.3938	0.9576	0.9473	0.9536	0.3436	0.3660
633	K562	KAP1	0.2033	0.9451	0.1972	0.9494	0.9252	0.9400	0.1579	0.2010
634	K562	MafF	0.4943	0.9755	0.4644	0.9729	0.9631	0.9555	0.3690	0.3668
635	K562	MafK	0.4860	0.9778	0.4510	0.9742	0.9650	0.9593	0.3444	0.3600
636	K562	Max	0.4268	0.9613	0.3983	0.9579	0.9485	0.9557	0.3604	0.3908
637	K562	MAZ	0.5070	0.9697	0.4810	0.9675	0.9604	0.9662	0.4506	0.4864
638	K562	Mxi1	0.3114	0.9778	0.2835	0.9745	0.9671	0.9727	0.1993	0.2402
639	K562	NELFe	0.1138	0.9713	0.0852	0.9730	0.9644	0.9733	0.0416	0.0861
640	K562	NF-E2	0.2775	0.9944	0.2530	0.9922	0.9780	0.9742	0.1569	0.1501
641	K562	NF-YA	0.4520	0.9944	0.4257	0.9899	0.9874	0.9923	0.3545	0.4179
642	K562	NF-YB	0.6031	0.9871	0.5894	0.9865	0.9847	0.9884	0.5119	0.5769
643	K562	Nrf1	0.4838	0.9845	0.5058	0.9845	0.9840	0.9862	0.3438	0.4230
644	K562	p300	0.2982	0.9457	0.2783	0.9424	0.9239	0.9316	0.2286	0.2471
645	K562	Pol2	0.5105	0.9695	0.4768	0.9679	0.9637	0.9676	0.4422	0.4807
646	K562	Pol2	0.5016	0.9702	0.4669	0.9649	0.9618	0.9662	0.4435	0.4758
647	K562	Pol2	0.4929	0.9657	0.4640	0.9637	0.9589	0.9650	0.4349	0.4741
648	K562	Pol2	0.5690	0.9674	0.5251	0.9649	0.9608	0.9648	0.4929	0.5347
649	K562	Pol2	0.1725	0.9338	0.1581	0.9289	0.9173	0.9245	0.1272	0.1463
650	K562	ol2(phosphoS2	0.1497	0.9180	0.0914	0.8954	0.8868	0.8928	0.0835	0.0899
651	K562	ol2(phosphoS2	0.0390	0.8714	0.0116	0.8308	0.7971	0.8120	0.0082	0.0132
652	K562	Pol2	0.5464	0.9704	0.5080	0.9672	0.9610	0.9653	0.4816	0.5152
653	K562	Pol3	0.0001	0.9771	0.0000	0.9333	0.8909	0.9403	0.0000	0.0000
654	K562	Rad21	0.6446	0.9961	0.6126	0.9955	0.9938	0.9947	0.5798	0.6072
655	K562	RFX5	0.0908	0.9100	0.0913	0.8865	0.8598	0.8914	0.0470	0.0704
656	K562	RPC155	0.1733	0.9509	0.1543	0.9490	0.9279	0.9389	0.0439	0.1064
657	K562	SETDB1	0.1333	0.9392	0.0842	0.9306	0.9067	0.9291	0.0795	0.1083
658	K562	SETDB1	0.1372	0.9680	0.1243	0.9640	0.9475	0.9564	0.0835	0.1209
659	K562	SIRT6	0.1393	0.9759	0.0982	0.9724	0.9617	0.9683	0.0714	0.1049
660	K562	SMC3	0.7040	0.9927	0.6700	0.9919	0.9889	0.9909	0.6373	0.6694
661	K562	STAT1	0.1242	0.9750	0.1196	0.9743	0.9540	0.9689	0.0532	0.1285
662	K562	STAT1	0.0819	0.9602	0.0516	0.9496	0.9479	0.9571	0.0324	0.0969
663	K562	STAT1	0.2038	0.9670	0.1980	0.9657	0.9438	0.9616	0.1132	0.1842

664	K562	STAT1	0.1393	0.9715	0.1100	0.9684	0.9390	0.9655	0.0731	0.1177
665	K562	STAT2	0.1461	0.9765	0.1112	0.9722	0.9630	0.9727	0.0622	0.1192
666	K562	STAT2	0.1157	0.9657	0.0817	0.9565	0.9459	0.9598	0.0425	0.1311
667	K562	TAL1	0.4219	0.9631	0.4155	0.9641	0.9561	0.9578	0.3196	0.3452
668	K562	TBLR1	0.1723	0.9462	0.1462	0.9458	0.9383	0.9433	0.0864	0.1326
669	K562	TBLR1	0.2585	0.9681	0.2163	0.9644	0.9575	0.9602	0.1816	0.2056
670	K562	TBP	0.4005	0.9543	0.3729	0.9510	0.9440	0.9505	0.3474	0.3731
671	K562	TFIIIC-110	0.0923	0.9380	0.0963	0.9310	0.8970	0.9116	0.0438	0.0806
672	K562	TR4	0.0814	0.9226	0.0600	0.9310	0.9374	0.9083	0.0235	0.0554
673	K562	UBF	0.2191	0.9798	0.2076	0.9809	0.9783	0.9817	0.1983	0.2211
674	K562	UBTF	0.2899	0.9678	0.2923	0.9655	0.9639	0.9657	0.2805	0.2861
675	K562	USF2	0.4625	0.9818	0.4461	0.9827	0.9808	0.9862	0.3883	0.4131
676	K562	YY1	0.5001	0.9931	0.4806	0.9933	0.9896	0.9906	0.4080	0.4192
677	K562	Znf143	0.6004	0.9631	0.5887	0.9626	0.9473	0.9565	0.5251	0.5777
678	K562	ZNF263	0.2783	0.9818	0.2379	0.9820	0.9581	0.9618	0.1363	0.1434
679	K562	ZNF274	0.4167	0.9433	0.3711	0.9368	0.9070	0.9114	0.2415	0.3359
680	K562	ZNF274	0.3977	0.9763	0.3783	0.9541	0.9558	0.9523	0.3355	0.4317
681	MCF10A-Er-Src	c-Fos	0.5633	0.9734	0.5560	0.9725	0.9638	0.9685	0.5084	0.5266
682	MCF10A-Er-Src	c-Fos	0.5997	0.9693	0.5950	0.9689	0.9599	0.9649	0.5488	0.5691
683	MCF10A-Er-Src	c-Fos	0.5744	0.9689	0.5665	0.9686	0.9585	0.9644	0.5166	0.5407
684	MCF10A-Er-Src	c-Fos	0.5981	0.9740	0.5923	0.9739	0.9670	0.9707	0.5529	0.5661
685	MCF10A-Er-Src	c-Myc	0.3967	0.9552	0.3769	0.9521	0.9356	0.9461	0.3153	0.3508

686	MCF10A-Er-Src	c-Myc	0.3813	0.9621	0.3550	0.9593	0.9431	0.9530	0.3019	0.3255
687	MCF10A-Er-Src	E2F4	0.3766	0.9597	0.3655	0.9569	0.9515	0.9579	0.3489	0.3611
688	MCF10A-Er-Src	Pol2	0.5278	0.9370	0.5003	0.9321	0.9198	0.9278	0.4771	0.4926
689	MCF10A-Er-Src	Pol2	0.5598	0.9479	0.5339	0.9435	0.9351	0.9401	0.5073	0.5294
690	MCF10A-Er-Src	STAT3	0.4373	0.9567	0.4215	0.9553	0.9385	0.9500	0.3591	0.3963
691	MCF10A-Er-Src	STAT3	0.4253	0.9550	0.4142	0.9538	0.9383	0.9482	0.3580	0.3880
692	MCF10A-Er-Src	STAT3	0.3046	0.9687	0.2821	0.9673	0.9488	0.9605	0.2245	0.2557
693	MCF10A-Er-Src	STAT3	0.4134	0.9564	0.4008	0.9556	0.9404	0.9494	0.3443	0.3750
694	MCF10A-Er-Src	STAT3	0.4741	0.9533	0.4631	0.9528	0.9354	0.9462	0.3814	0.4260
695	MCF-7	GATA3	0.2138	0.9185	0.2218	0.9227	0.8827	0.8808	0.1338	0.1439
696	MCF-7	GATA3	0.1322	0.9220	0.1309	0.9246	0.8844	0.8885	0.0738	0.0731
697	MCF-7	HA-E2F1	0.4755	0.9604	0.4577	0.9573	0.9486	0.9566	0.4420	0.4640
698	MCF-7	TCF7L2	0.1545	0.8939	0.1453	0.8955	0.8543	0.8751	0.0935	0.1234
699	MCF-7	ZNF217	0.2308	0.9077	0.2158	0.9098	0.8451	0.8879	0.1105	0.1670
700	NB4	c-Myc	0.4088	0.9641	0.3746	0.9605	0.9511	0.9593	0.3153	0.3646
701	NB4	Max	0.4271	0.9625	0.4071	0.9600	0.9495	0.9586	0.3450	0.3956

702	NB4	Pol2	0.5565	0.9767	0.5327	0.9765	0.9745	0.9758	0.5051	0.5361
703	NT2-D1	SUZ12	0.2642	0.9758	0.2327	0.9729	0.9681	0.9723	0.2079	0.2428
704	NT2-D1	YY1	0.4335	0.9867	0.4314	0.9873	0.9784	0.9830	0.3141	0.3637
705	NT2-D1	ZNF274	0.3552	0.9347	0.3086	0.9191	0.9162	0.9249	0.3035	0.3448
706	PANC-1	TCF7L2	0.1104	0.8777	0.1041	0.8739	0.8487	0.8598	0.0822	0.0947
707	PBDEFetal	GATA1	0.3241	0.9787	0.2771	0.9815	0.9780	0.9793	0.1532	0.1885
708	PBDE	GATA1	0.3548	0.9397	0.3364	0.9366	0.9159	0.9275	0.2615	0.3048
709	PBDE	Pol2	0.5676	0.9604	0.5358	0.9576	0.9527	0.9577	0.5011	0.5300
710	Raji	Pol2	0.6125	0.9588	0.5749	0.9556	0.9515	0.9506	0.5563	0.5759
711	SH-SY5Y	GATA2	0.3984	0.9390	0.4064	0.9408	0.9176	0.9140	0.3070	0.3089
712	SH-SY5Y	GATA3	0.2830	0.9445	0.2922	0.9473	0.9228	0.9237	0.2066	0.2195
713	U2OS	KAP1	0.1824	0.8927	0.1537	0.8832	0.8476	0.8716	0.1209	0.1453
714	U2OS	SETDB1	0.4381	0.9213	0.4291	0.9136	0.8917	0.9056	0.3540	0.4152
715	K562	eGFP-FOS	0.3878	0.9837	0.3597	0.9837	0.9804	0.9820	0.3182	0.3384
716	K562	eGFP-GATA2	0.2641	0.9535	0.2342	0.9509	0.9338	0.9378	0.1875	0.2026
717	K562	eGFP-HDAC8	0.0308	0.8959	0.0317	0.9018	0.8859	0.8964	0.0205	0.0245
718	K562	eGFP-JunB	0.3020	0.9686	0.2791	0.9653	0.9593	0.9629	0.2309	0.2645
719	K562	eGFP-JunD	0.3377	0.9414	0.3109	0.9368	0.9190	0.9278	0.2650	0.2934
720	A549	CTCF	0.6926	0.9863	0.6917	0.9869	0.9794	0.9852	0.6344	0.6823
721	A549	Pol2	0.5352	0.9862	0.5079	0.9847	0.9835	0.9846	0.4758	0.5014
722	Fibrobl	CTCF	0.6441	0.9872	0.6361	0.9868	0.9817	0.9859	0.5880	0.6286
723	Gliobla	CTCF	0.6981	0.9847	0.6943	0.9848	0.9792	0.9835	0.6534	0.6824
724	Gliobla	Pol2	0.5081	0.9857	0.4860	0.9836	0.9822	0.9831	0.4484	0.4798
725	GM12878	c-Myc	0.3692	0.9837	0.3421	0.9824	0.9815	0.9822	0.2549	0.3050
726	GM12878	CTCF	0.6648	0.9895	0.6560	0.9898	0.9855	0.9889	0.6122	0.6454
727	GM12878	Pol2	0.4723	0.9479	0.4504	0.9437	0.9426	0.9458	0.4204	0.4439
728	GM12891	CTCF	0.6321	0.9879	0.6265	0.9876	0.9820	0.9869	0.5650	0.6192

729	GM12892	CTCF	0.6183	0.9846	0.6143	0.9845	0.9769	0.9838	0.5557	0.6069
730	GM19238	CTCF	0.6581	0.9849	0.6543	0.9847	0.9771	0.9837	0.5913	0.6449
731	GM19239	CTCF	0.5873	0.9863	0.5885	0.9860	0.9812	0.9854	0.5229	0.5787
732	GM19240	CTCF	0.6722	0.9881	0.6664	0.9881	0.9823	0.9873	0.6133	0.6616
733	H1-hESC	c-Myc	0.0278	0.9196	0.0198	0.9186	0.9123	0.9195	0.0251	0.0226
734	H1-hESC	CTCF	0.6505	0.9915	0.6428	0.9914	0.9868	0.9908	0.5461	0.6244
735	H1-hESC	Pol2	0.5045	0.9654	0.4819	0.9628	0.9552	0.9604	0.4525	0.4678
736	HeLa-S3	c-Myc	0.1627	0.9686	0.1468	0.9692	0.9538	0.9616	0.0947	0.1198
737	HeLa-S3	CTCF	0.6499	0.9884	0.6356	0.9884	0.9834	0.9871	0.5878	0.6287
738	HeLa-S3	Pol2	0.5568	0.9568	0.5194	0.9520	0.9490	0.9528	0.4991	0.5327
739	HepG2	c-Myc	0.3033	0.9887	0.2593	0.9875	0.9855	0.9876	0.1468	0.2003
740	HepG2	CTCF	0.7910	0.9963	0.7752	0.9961	0.9939	0.9955	0.7348	0.7642
741	HepG2	Pol2	0.4316	0.9503	0.4152	0.9395	0.9358	0.9422	0.3929	0.4074
742	HUVEC	c-Myc	0.2575	0.9877	0.2317	0.9843	0.9774	0.9812	0.1705	0.1972
743	HUVEC	CTCF	0.6056	0.9891	0.5977	0.9895	0.9854	0.9881	0.5559	0.5888
744	HUVEC	Pol2	0.5321	0.9835	0.5075	0.9799	0.9779	0.9789	0.4552	0.5011
745	K562	c-Myc	0.2824	0.9828	0.2683	0.9818	0.9785	0.9819	0.2043	0.2465
746	K562	CTCF	0.6459	0.9898	0.6352	0.9893	0.9853	0.9883	0.5938	0.6289
747	K562	Pol2	0.4967	0.9582	0.4607	0.9546	0.9482	0.9546	0.4314	0.4644
748	MCF-7	c-Myc	0.2730	0.9638	0.2588	0.9649	0.9544	0.9628	0.2040	0.2385
749	MCF-7	c-Myc	0.3959	0.9743	0.3852	0.9730	0.9691	0.9723	0.3539	0.3769
750	MCF-7	c-Myc	0.1939	0.9681	0.1862	0.9678	0.9648	0.9679	0.1435	0.1780
751	MCF-7	c-Myc	0.4209	0.9636	0.4058	0.9621	0.9495	0.9595	0.3586	0.3953
752	MCF-7	CTCF	0.6681	0.9859	0.6608	0.9857	0.9809	0.9847	0.5853	0.6521

753	MCF-7	CTCF	0.6483	0.9842	0.6511	0.9843	0.9800	0.9836	0.6039	0.6442
754	MCF-7	CTCF	0.6306	0.9830	0.6307	0.9834	0.9782	0.9826	0.5830	0.6232
755	MCF-7	CTCF	0.6510	0.9852	0.6516	0.9852	0.9794	0.9844	0.5993	0.6440
756	MCF-7	CTCF	0.6938	0.9829	0.6938	0.9830	0.9759	0.9811	0.6430	0.6834
757	MCF-7	Pol2	0.6030	0.9772	0.5756	0.9752	0.9747	0.9761	0.5538	0.5817
758	MCF-7	Pol2	0.5304	0.9744	0.5088	0.9732	0.9722	0.9743	0.4715	0.5028
759	MCF-7	Pol2	0.4375	0.9433	0.4118	0.9353	0.9298	0.9351	0.3924	0.4149
760	NHEK	CTCF	0.6861	0.9872	0.6830	0.9872	0.9806	0.9863	0.6232	0.6681
761	ProgFib	CTCF	0.6378	0.9891	0.6148	0.9886	0.9836	0.9877	0.5625	0.6151
762	ProgFib	Pol2	0.4746	0.9602	0.4464	0.9551	0.9515	0.9540	0.4244	0.4518
763	A549	CTCF	0.7154	0.9860	0.7053	0.9859	0.9797	0.9835	0.6437	0.6889
764	AG04449	CTCF	0.6826	0.9885	0.6607	0.9883	0.9837	0.9864	0.6185	0.6575
765	AG04450	CTCF	0.6896	0.9844	0.6661	0.9836	0.9764	0.9803	0.6190	0.6547
766	AG09309	CTCF	0.7364	0.9884	0.7123	0.9879	0.9827	0.9863	0.6633	0.7028
767	AG09319	CTCF	0.7069	0.9842	0.6915	0.9838	0.9783	0.9817	0.6486	0.6811
768	AG10803	CTCF	0.6805	0.9842	0.6626	0.9839	0.9774	0.9815	0.6205	0.6498
769	AoAF	CTCF	0.6646	0.9863	0.6489	0.9854	0.9794	0.9831	0.5986	0.6359
770	BE2_C	CTCF	0.6975	0.9814	0.6993	0.9815	0.9748	0.9796	0.6630	0.6894
771	BJ	CTCF	0.7150	0.9848	0.6981	0.9839	0.9779	0.9814	0.6567	0.6878
772	Caco-2	CTCF	0.7378	0.9919	0.7264	0.9917	0.9883	0.9908	0.6859	0.7103
773	GM06990	CTCF	0.7046	0.9870	0.7004	0.9867	0.9815	0.9852	0.6523	0.6889
774	GM12801	CTCF	0.3812	0.9975	0.3361	0.9972	0.9964	0.9953	0.2625	0.3311
775	GM12864	CTCF	0.7171	0.9870	0.7078	0.9864	0.9815	0.9847	0.6590	0.6918
776	GM12865	CTCF	0.7297	0.9892	0.7176	0.9883	0.9839	0.9871	0.6762	0.7075

777	GM12872	CTCF	0.7176	0.9872	0.7096	0.9865	0.9815	0.9844	0.6607	0.6913
778	GM12873	CTCF	0.7291	0.9859	0.7181	0.9851	0.9786	0.9832	0.6683	0.7048
779	GM12874	CTCF	0.7253	0.9919	0.7118	0.9915	0.9884	0.9902	0.6628	0.6996
780	GM12875	CTCF	0.7244	0.9911	0.7142	0.9905	0.9870	0.9892	0.6617	0.6979
781	GM12878	CTCF	0.7361	0.9913	0.7236	0.9910	0.9874	0.9893	0.6692	0.7092
782	HAc	CTCF	0.7056	0.9830	0.6911	0.9823	0.9756	0.9798	0.6442	0.6786
783	HA-sp	CTCF	0.7201	0.9884	0.7074	0.9879	0.9835	0.9864	0.6661	0.7014
784	HBMEC	CTCF	0.7133	0.9833	0.7046	0.9829	0.9757	0.9800	0.6550	0.6903
785	HCFaa	CTCF	0.7041	0.9887	0.6869	0.9878	0.9833	0.9862	0.6403	0.6730
786	HCM	CTCF	0.7180	0.9811	0.7032	0.9809	0.9736	0.9784	0.6579	0.6910
787	HCPEpiC	CTCF	0.7082	0.9813	0.6992	0.9808	0.9733	0.9772	0.6538	0.6826
788	HCT-116	CTCF	0.7130	0.9872	0.7022	0.9864	0.9806	0.9843	0.6560	0.6873
789	HEEpiC	CTCF	0.7040	0.9848	0.6858	0.9838	0.9774	0.9821	0.6366	0.6737
790	HEK293	CTCF	0.7162	0.9898	0.7045	0.9893	0.9852	0.9885	0.6535	0.6900
791	HeLa-S3	CTCF	0.7086	0.9903	0.6953	0.9899	0.9861	0.9887	0.6476	0.6842
792	HepG2	CTCF	0.7144	0.9906	0.7085	0.9902	0.9858	0.9886	0.6458	0.6981
793	HFF	CTCF	0.7176	0.9904	0.6914	0.9896	0.9854	0.9879	0.6503	0.6807
794	HFF-Myc	CTCF	0.7371	0.9862	0.7222	0.9857	0.9798	0.9832	0.6721	0.7058
795	HL-60	CTCF	0.7410	0.9968	0.7235	0.9966	0.9953	0.9965	0.6720	0.7162
796	HMEC	CTCF	0.7117	0.9846	0.7011	0.9839	0.9765	0.9821	0.6453	0.6846
797	HMF	CTCF	0.7076	0.9855	0.6926	0.9849	0.9793	0.9824	0.6428	0.6790
798	HPAF	CTCF	0.7135	0.9820	0.7034	0.9817	0.9746	0.9786	0.6570	0.6892
799	HPF	CTCF	0.6899	0.9867	0.6698	0.9859	0.9807	0.9838	0.6254	0.6579
800	HRE	CTCF	0.7163	0.9880	0.7044	0.9879	0.9831	0.9862	0.6547	0.6899

801	HRPEpiC	CTCF	0.7112	0.9874	0.7060	0.9873	0.9830	0.9856	0.6555	0.6915
802	HUVEC	CTCF	0.7243	0.9909	0.7115	0.9906	0.9873	0.9893	0.6686	0.6989
803	HVMF	CTCF	0.7141	0.9854	0.7025	0.9846	0.9786	0.9826	0.6557	0.6920
804	K562	CTCF	0.7008	0.9899	0.6914	0.9894	0.9853	0.9880	0.6362	0.6786
805	MCF-7	CTCF	0.6846	0.9853	0.6761	0.9848	0.9793	0.9830	0.6317	0.6685
806	NB4	CTCF	0.7330	0.9928	0.7220	0.9925	0.9897	0.9912	0.6775	0.7123
807	NHDF-neo	CTCF	0.6864	0.9809	0.6664	0.9804	0.9733	0.9775	0.6278	0.6574
808	NHEK	CTCF	0.7168	0.9872	0.7039	0.9867	0.9806	0.9847	0.6512	0.6939
809	NHLF	CTCF	0.6706	0.9873	0.6474	0.9866	0.9812	0.9848	0.5987	0.6362
810	RPTEC	CTCF	0.7529	0.9835	0.7443	0.9827	0.9762	0.9809	0.7019	0.7354
811	SAEC	CTCF	0.7345	0.9899	0.7183	0.9892	0.9851	0.9875	0.6749	0.7048
812	SK-N-SH_RA	CTCF	0.7241	0.9908	0.7134	0.9906	0.9872	0.9894	0.6761	0.7010
813	WERI-Rb-1	CTCF	0.6908	0.9894	0.6880	0.9894	0.9848	0.9882	0.6258	0.6700
814	WI-38	CTCF	0.7332	0.9474	0.7263	0.9464	0.9344	0.9415	0.6888	0.7131
815	H1-hESC	H2AK5ac	0.3687	0.8616	0.3586	0.8552	0.8526	0.8544	0.3478	0.3582
816	H1-hESC	H2AZ	0.1750	0.8855	0.1669	0.8818	0.8800	0.8832	0.1617	0.1694
817	H1-hESC	H2BK120ac	0.0897	0.8170	0.0869	0.8138	0.7993	0.8106	0.0559	0.0697
818	H1-hESC	H2BK12ac	0.1867	0.8659	0.1770	0.8612	0.8579	0.8624	0.1616	0.1706
819	H1-hESC	H2BK15ac	0.1259	0.8869	0.1198	0.8855	0.8874	0.8877	0.1234	0.1196
820	H1-hESC	H2BK20ac	0.0803	0.8407	0.0752	0.8377	0.8249	0.8301	0.0557	0.0612
821	H1-hESC	H2BK5ac	0.2733	0.8701	0.2650	0.8686	0.8543	0.8626	0.2331	0.2468
822	H1-hESC	H3K14ac	0.0785	0.8989	0.0774	0.8958	0.8876	0.8948	0.0677	0.0732

823	H1-hESC	H3K18ac	0.3558	0.8691	0.3474	0.8657	0.8554	0.8603	0.3220	0.3347
824	H1-hESC	H3K23ac	0.0475	0.8733	0.0493	0.8697	0.8707	0.8727	0.0504	0.0507
825	H1-hESC	H3K23me2	0.5467	0.9599	0.5335	0.9581	0.9558	0.9583	0.5097	0.5300
826	H1-hESC	H3K27ac	0.4604	0.8763	0.4445	0.8713	0.8558	0.8652	0.4040	0.4300
827	H1-hESC	H3K27me3	0.5163	0.8938	0.4763	0.8843	0.8793	0.8835	0.4456	0.4684
828	H1-hESC	H3K36me3	0.3576	0.8749	0.2104	0.8081	0.7898	0.8030	0.1846	0.2095
829	H1-hESC	H3K4ac	0.0752	0.8723	0.0708	0.8701	0.8568	0.8666	0.0535	0.0622
830	H1-hESC	H3K4me1	0.4048	0.8684	0.3932	0.8622	0.8487	0.8569	0.3559	0.3772
831	H1-hESC	H3K4me2	0.7663	0.9295	0.7500	0.9225	0.9099	0.9175	0.7210	0.7404
832	H1-hESC	H3K4me3	0.9049	0.9838	0.8834	0.9779	0.9759	0.9778	0.8742	0.8831
833	H1-hESC	H3K56ac	0.3385	0.9081	0.3303	0.9085	0.8995	0.9057	0.2986	0.3210
834	H1-hESC	H3K79me1	0.1446	0.8992	0.1174	0.8791	0.8755	0.8789	0.1162	0.1190
835	H1-hESC	H3K79me2	0.1692	0.8838	0.1273	0.8568	0.8500	0.8565	0.1246	0.1297
836	H1-hESC	H3K9ac	0.6982	0.9515	0.6663	0.9446	0.9407	0.9433	0.6483	0.6632
837	H1-hESC	H3K9me3	0.2889	0.8611	0.2728	0.8382	0.8227	0.8321	0.2276	0.2586
838	H1-hESC	H4K20me1	0.2823	0.8869	0.2599	0.8699	0.8676	0.8708	0.2555	0.2605
839	H1-hESC	H4K5ac	0.3646	0.8786	0.3530	0.8761	0.8589	0.8691	0.3148	0.3368

840	H1-hESC	H4K8ac	0.4946	0.9205	0.4891	0.9196	0.9195	0.9202	0.4849	0.4895
841	H1-hESC	H4K91ac	0.5645	0.8605	0.5323	0.8479	0.8322	0.8411	0.5014	0.5203
842	K562	H2AZ	0.5108	0.8833	0.4824	0.8747	0.8606	0.8681	0.4558	0.4756
843	K562	H3K27ac	0.5352	0.8826	0.4932	0.8716	0.8563	0.8667	0.4622	0.4864
844	K562	H3K27me3	0.1036	0.8620	0.0965	0.8545	0.8526	0.8561	0.0983	0.0993
845	K562	H3K36me3	0.2696	0.8545	0.1648	0.7978	0.7879	0.7968	0.1426	0.1593
846	K562	H3K4me1	0.4096	0.8308	0.3767	0.8160	0.7971	0.8080	0.3361	0.3576
847	K562	H3K4me2	0.6287	0.9043	0.5881	0.8918	0.8785	0.8870	0.5601	0.5822
848	K562	H3K4me3	0.6518	0.9200	0.6046	0.9065	0.8951	0.9036	0.5825	0.6046
849	K562	H3K79me2	0.4364	0.8576	0.3564	0.8151	0.8089	0.8160	0.3381	0.3560
850	K562	H3K9ac	0.6018	0.9061	0.5506	0.8922	0.8808	0.8890	0.5270	0.5519
851	K562	H3K9me1	0.0512	0.8825	0.0469	0.8759	0.8795	0.8818	0.0451	0.0479
852	K562	H3K9me3	0.0968	0.8784	0.0775	0.8729	0.8526	0.8650	0.0697	0.0835
853	K562	H4K20me1	0.1837	0.8941	0.1407	0.8703	0.8665	0.8703	0.1391	0.1511
854	Monocytes-CD14+RO01746??	H2AZ	0.6242	0.8985	0.6030	0.8908	0.8823	0.8888	0.5818	0.5995
855	Monocytes-CD14+RO01746??	H3K27ac	0.5407	0.8374	0.4909	0.8096	0.7973	0.8061	0.4659	0.4867
856	Monocytes-CD14+RO01746??	H3K27me3	0.4804	0.8224	0.4463	0.8040	0.7949	0.8012	0.4262	0.4440

857	Monocytes-CD14+RO01746??	H3K36me3	0.4287	0.8474	0.2764	0.7694	0.7570	0.7670	0.2541	0.2744
858	Monocytes-CD14+RO01746??	H3K4me1	0.5921	0.8397	0.5546	0.8227	0.8072	0.8168	0.5184	0.5441
859	Monocytes-CD14+RO01746??	H3K4me2	0.7324	0.9193	0.7015	0.9076	0.8966	0.9030	0.6746	0.6952
860	Monocytes-CD14+RO01746??	H3K4me3	0.6983	0.9087	0.6684	0.8985	0.8865	0.8939	0.6388	0.6625
861	Monocytes-CD14+RO01746??	H3K79me2	0.4023	0.8342	0.3187	0.7755	0.7694	0.7761	0.3034	0.3190
862	Monocytes-CD14+RO01746??	H3K9ac	0.6576	0.9245	0.6255	0.9146	0.9090	0.9133	0.6039	0.6243

863	Monocytes-CD14+RO01746??	H3K9me3	0.3665	0.8562	0.3411	0.8336	0.8193	0.8326	0.2922	0.3337
864	Monocytes-CD14+RO01746??	H4K20me1	0.3553	0.8264	0.2669	0.7553	0.7521	0.7578	0.2641	0.2707
865	NH-A	H2AZ	0.6902	0.9463	0.6686	0.9418	0.9345	0.9392	0.6437	0.6630
866	NH-A	H3K27ac	0.5981	0.8751	0.5623	0.8624	0.8483	0.8542	0.5303	0.5464
867	NH-A	H3K27me3	0.3472	0.8553	0.3075	0.8365	0.8291	0.8369	0.2841	0.3034
868	NH-A	H3K36me3	0.2987	0.8626	0.1624	0.7894	0.7750	0.7893	0.1339	0.1547
869	NH-A	H3K4me1	0.5832	0.8533	0.5563	0.8391	0.8217	0.8305	0.5183	0.5381
870	NH-A	H3K4me2	0.7578	0.9124	0.7313	0.9004	0.8865	0.8932	0.7004	0.7176
871	NH-A	H3K4me3	0.7576	0.9443	0.7232	0.9323	0.9270	0.9313	0.7080	0.7216
872	NH-A	H3K79me2	0.4307	0.8499	0.3514	0.7971	0.7928	0.7981	0.3358	0.3504
873	NH-A	H3K9ac	0.6685	0.9061	0.6295	0.8919	0.8814	0.8875	0.6024	0.6212
874	NH-A	H3K9me3	0.1929	0.8516	0.1802	0.8383	0.8258	0.8338	0.1520	0.1735
875	NH-A	H4K20me1	0.3342	0.8583	0.2567	0.8062	0.8026	0.8079	0.2510	0.2570
876	NHDF-Ad	H2AZ	0.6151	0.9441	0.5851	0.9392	0.9313	0.9367	0.5558	0.5793
877	NHDF-Ad	H3K27ac	0.4761	0.8780	0.4304	0.8614	0.8501	0.8555	0.4033	0.4214
878	NHDF-Ad	H3K27me3	0.2665	0.8864	0.2318	0.8702	0.8636	0.8713	0.2013	0.2229
879	NHDF-Ad	H3K36me3	0.1446	0.8754	0.0673	0.8098	0.7932	0.8064	0.0501	0.0638
880	NHDF-Ad	H3K4me1	0.3995	0.8290	0.3672	0.8120	0.7971	0.8058	0.3359	0.3515
881	NHDF-Ad	H3K4me2	0.6565	0.9133	0.6139	0.8972	0.8836	0.8921	0.5810	0.6034

882	NHDF-Ad	H3K4me3	0.7254	0.9584	0.6727	0.9458	0.9421	0.9462	0.6537	0.6742
883	NHDF-Ad	H3K79me2	0.3675	0.8547	0.2889	0.7989	0.7933	0.7983	0.2741	0.2834
884	NHDF-Ad	H3K9ac	0.6438	0.9403	0.5864	0.9263	0.9198	0.9247	0.5656	0.5887
885	NHDF-Ad	H3K9me3	0.1417	0.9009	0.1105	0.8887	0.8641	0.8777	0.0942	0.1247
886	NHDF-Ad	H4K20me1	0.2079	0.8444	0.1596	0.8095	0.8069	0.8117	0.1643	0.1658
887	NHEK	H2AZ	0.5650	0.9051	0.5401	0.8988	0.8857	0.8957	0.5080	0.5325
888	NHEK	H3K27ac	0.5908	0.8710	0.5444	0.8534	0.8343	0.8483	0.5051	0.5331
889	NHEK	H3K27me3	0.4429	0.8823	0.4041	0.8717	0.8634	0.8697	0.3753	0.3953
890	NHEK	H3K36me3	0.3474	0.8570	0.2362	0.7983	0.7921	0.8000	0.2167	0.2375
891	NHEK	H3K4me1	0.5627	0.8368	0.5284	0.8206	0.7930	0.8132	0.4688	0.5122
892	NHEK	H3K4me2	0.6864	0.8933	0.6507	0.8783	0.8576	0.8736	0.6084	0.6412
893	NHEK	H3K4me3	0.7399	0.9356	0.7070	0.9234	0.9129	0.9216	0.6843	0.7047
894	NHEK	H3K79me2	0.4482	0.8559	0.3573	0.7986	0.7945	0.8004	0.3432	0.3561
895	NHEK	H3K9ac	0.6631	0.9127	0.6178	0.8971	0.8852	0.8936	0.5894	0.6120
896	NHEK	H3K9me1	0.0762	0.8567	0.0593	0.8263	0.8256	0.8273	0.0578	0.0602
897	NHEK	H3K9me3	0.1942	0.8638	0.1744	0.8284	0.8167	0.8259	0.1414	0.1702
898	NHEK	H4K20me1	0.1608	0.8525	0.1082	0.8120	0.8077	0.8154	0.1031	0.1111
899	NHLF	H2AZ	0.5793	0.9412	0.5469	0.9346	0.9270	0.9336	0.5162	0.5419
900	NHLF	H3K27ac	0.5758	0.8813	0.5323	0.8643	0.8519	0.8586	0.5027	0.5222
901	NHLF	H3K27me3	0.3313	0.8704	0.2929	0.8551	0.8495	0.8560	0.2692	0.2911
902	NHLF	H3K36me3	0.3274	0.9232	0.2211	0.8875	0.8812	0.8878	0.2065	0.2270
903	NHLF	H3K4me1	0.3393	0.8289	0.3108	0.8097	0.7959	0.8055	0.2844	0.3002
904	NHLF	H3K4me2	0.6433	0.9090	0.5960	0.8903	0.8787	0.8870	0.5693	0.5893
905	NHLF	H3K4me3	0.7916	0.9699	0.7407	0.9572	0.9547	0.9582	0.7267	0.7462
906	NHLF	H3K79me2	0.4068	0.8597	0.3225	0.8026	0.8001	0.8062	0.3086	0.3237
907	NHLF	H3K9ac	0.7181	0.9580	0.6781	0.9495	0.9468	0.9496	0.6639	0.6822
908	NHLF	H3K9me3	0.1821	0.9311	0.1650	0.9089	0.9048	0.9126	0.1637	0.1930
909	NHLF	H4K20me1	0.0518	0.8481	0.0290	0.8059	0.7934	0.8059	0.0245	0.0278

910	Osteoblasts	H2AZ	0.5943	0.9038	0.5660	0.8968	0.8864	0.8940	0.5396	0.5602
911	Osteoblasts	H3K27ac	0.5700	0.8417	0.5286	0.8229	0.8075	0.8151	0.4985	0.5139
912	Osteoblasts	H3K27me3	0.3157	0.8121	0.2878	0.7927	0.7830	0.7921	0.2711	0.2852
913	Osteoblasts	H3K36me3	0.3637	0.8453	0.2282	0.7808	0.7706	0.7810	0.2015	0.2222
914	Osteoblasts	H3K4me1	0.5746	0.8313	0.5392	0.8136	0.7932	0.8036	0.5010	0.5200
915	Osteoblasts	H3K4me2	0.7058	0.9004	0.6691	0.8854	0.8690	0.8786	0.6366	0.6561
916	Osteoblasts	H3K4me3	0.7370	0.9354	0.6999	0.9224	0.9139	0.9204	0.6801	0.6973
917	Osteoblasts	H3K79me2	0.4349	0.8439	0.3516	0.7880	0.7837	0.7899	0.3386	0.3520
918	Osteoblasts	H3K9me3	0.1550	0.8527	0.1340	0.8199	0.8073	0.8165	0.1037	0.1299
		**Average**	**0.4103**	**0.9481**	**0.3905**	**0.9428**	**0.9326**	**0.9384**	**0.3425**	**0.3709**
